# Piezo1‐Mediated Mechanotransduction Contributes to Disturbed Flow‐Induced Atherosclerotic Endothelial Inflammation

**DOI:** 10.1161/JAHA.123.035558

**Published:** 2024-10-25

**Authors:** Yining Lan, Jing Lu, Shaohan Zhang, Chunxiao Jie, Chunyong Chen, Chao Xiao, Chao Qin, Daobin Cheng

**Affiliations:** ^1^ Department of Neurology The First Affiliated Hospital of Guangxi Medical University Nanning Guangxi China; ^2^ The Second Affiliated Hospital of Qiqihar Medical College Qiqihar Heilongjiang China; ^3^ Department of Neurology Liuzhou People’s Hospital Liuzhou Guangxi China

**Keywords:** atherosclerosis, endothelial cells, inflammation, Piezo1, shear stress, Atherosclerosis, Inflammation, Hemodynamics, Mechanisms, Vascular Biology

## Abstract

**Background:**

Disturbed flow generates oscillatory shear stress (OSS), which in turn leads to endothelial inflammation and atherosclerosis. Piezo1, a biomechanical force sensor, plays a crucial role in the cardiovascular system. However, the specific role of Piezo1 in atherosclerosis remains to be fully elucidated.

**Methods and Results:**

We detected the expression of Piezo1 in atherosclerotic mice and endothelial cells from regions with disturbed blood flow. The pharmacological inhibitor Piezo1 inhibitor (GsMTx4) was used to evaluate the impact of Piezo1 on plaque progression and endothelial inflammation. We examined Piezo1's direct response to OSS in vitro and its effects on endothelial inflammation. Furthermore, mechanistic studies were conducted to explore the potential molecular cascade through which Piezo1 mediates endothelial inflammation in response to OSS. Our findings revealed the upregulation of Piezo1 in apoE−/− (apolipoprotein E) atherosclerotic mice, which is associated with disturbed flow. Treatment with GsMTx4 not only delayed plaque progression but also mitigated endothelial inflammation in both chronic and disturbed flow‐induced atherosclerosis. Piezo1 was shown to facilitate calcium ions (Ca^2^^+^) influx in response to OSS, thereby activating endothelial inflammation. This inflammatory response was attenuated in the absence of Piezo1. Additionally, we identified that under OSS, Piezo1 activates the Ca^2^^+^/CaM/CaMKII (calmodulin/calmodulin‐dependent protein kinases Ⅱ) pathways, which subsequently stimulate downstream kinases FAK (focal adhesion kinase) and Src. This leads to the activation of the OSS‐sensitive YAP (yes‐associated protein), ultimately triggering endothelial inflammation.

**Conclusions:**

Our study highlights the key role of Piezo1 in atherosclerotic endothelial inflammation, proposing the Piezo1–Ca^2+^/CaM/CaMKII‐FAK/Src‐YAP axis as a previously unknown endothelial mechanotransduction pathway. Piezo1 is expected to become a potential therapeutic target for atherosclerosis and cardiovascular diseases.

**Nonstandard Abbreviations and Acronyms**
CaM: calmodulin
CaMKII: calcium/calmodulin‐dependent protein kinase II
DF: disturbed flow
EC: endothelial cell
FAK: focal adhesion kinase
HUVEC: human umbilical vein endothelial cell
ICAM‐1: intercellular adhesion molecule 1
LSS: laminar shear stress
OSS: oscillatory shear stress
VCAM‐1: vascular cell adhesion molecule 1
YAP: yes‐associated protein

**Research Perspective**

**What Is New?**

- This study elucidates the previously unknown role of Piezo1 in atherosclerotic endothelial inflammation, revealing the Piezo1–Ca^2+^/CaM/CaMKII‐FAK/Src‐YAP axis as a novel mechanotransduction pathway.

**What Question Should Be Addressed Next?**

- Future research should focus on further delineating the detailed molecular mechanisms underlying Piezo1‐mediated endothelial inflammation in response to oscillatory shear stress.
- The clinical efficacy and safety of drugs targeting Piezo1 should be developed and evaluated to create new therapies aimed at delaying the progression of human plaques and reducing the occurrence of cardiovascular events.

Closely associated with myocardial infarction and stroke, atherosclerosis is considered a chronic inflammatory disease that begins with the activation of endothelial cells (ECs).^1^ Blood shear stress refers to the friction exerted by the flowing blood on the surface of blood vessels.^2^ Laminar shear stress (LSS) generated by unidirectional flow in the straight region of the arterial tree is antiatherogenic, whereas oscillatory shear stress (OSS) generated by disturbed flow (DF) at arterial branches or bends is atherogenic. If exposed to DF for a long time, the expression of EC inflammatory cytokines, especially VCAM‐1 (vascular cell adhesion molecule 1) and ICAM‐1 (intercellular adhesion molecule 1), will increase. Inflammatory activation of ECs eventually advances and accelerates the development of atherosclerosis.^3^, ^4^, ^5^

Piezo1, a newly identified mechanosensitive protein located on the cell membrane, is capable of transducing mechanical signals into biological responses.^6^, ^7^ The wide expression of Piezo1 endows ECs with timely response to flow shear stress stimulation, which is a key link in various physiological and pathological processes of blood vessels.^8^, ^9^ Our comprehension of Piezo1's role in vascular biology commenced with 2 studies in 2014.^10^, ^11^ The reports confirmed that Piezo1 was expressed in vascular endothelium, and the knockout of the Piezo1 gene in mice is lethal to the embryos, as manifested by the defect of vascular development in embryonic mice and impaired endothelial response to blood flow. Subsequently, several studies have reported the key role of Piezo1 in maintaining vascular homeostasis in response to laminar shear stress.^12^, ^13^, ^14^, ^15^ However, little is known about Piezo1's biomechanical sensing role in the cardiovascular system, especially in atherosclerosis, which is closely related to shear stress of blood flow.

In this study, we evaluated the role of Piezo1 in atherosclerosis in vivo. We revealed that Piezo1 is upregulated in atherosclerotic plaque tissue, particularly in ECs exposed to proatherosclerotic DF. By using the pharmacological inhibitor GsMTx4, we observed the anti‐inflammatory and antiatherosclerotic effects of inhibiting Piezo1. In vitro, Piezo1 directly sensed the stimulation of OSS, mediating the influx of Ca^2+^ and activating endothelial inflammation. Mechanistically, Piezo1 activated the kinases FAK (focal adhesion kinase) and Src through the Ca^2+^/CaM/CaMKII pathway, leading to the dephosphorylation at the S127 site and nuclear translocation of YAP (yes‐associated protein), ultimately triggering endothelial inflammation.

## METHODS

### Data Availability

Most of the data underpinning this study are provided in the article and supplemental material. Reasonable requests for data access can be made by contacting the corresponding author.

### Antibodies and Reagents

For detailed information about the antibodies, reagents, and materials used in the experiments, please see Tables S1 through S4.

### Animals

All animal experiments were conducted in accordance with the National Institutes of Health *Guide for the Care and Use of Laboratory Animals*. The design and data collection of the animal studies adhered to the principles outlined in the ARRIVE guidelines (Animal Research: Reporting of In Vivo Experiments). All protocols involving animals were approved by the Laboratory Animal Welfare and Ethics Committee of Guangxi Medical University (Guangxi, China). Eight‐week‐old male C57BL/6J mice and apoE (apolipoprotein E)−/− mice were from the Nanjing Junke Bioengineering Corporation (Nanjing, China). All apoE−/− mice were fed a high‐fat Western diet (HFHC; Dyets, Bethlehem, PA). C57BL/6J mice were provided with a standard diet. All mice were housed in specific pathogen‐free animal rooms. They had unrestricted access to food and water and were maintained at a constant temperature (21 °C±1 °C) and a 12‐hour light/12‐hour dark cycle.

### Partial Carotid Artery Ligation

To establish a DF atherosclerosis model, left partial carotid artery ligation was conducted in apoE−/− mice.^16^ In brief, mice were anesthetized with 2% to 3% isoflurane. An incision was made in the middle of the neck to expose and separate the left common carotid artery and its 4 branches. Except for the superior thyroid artery, the other 3 arteries (external carotid, internal carotid, and occipital artery) were collectively ligated with 6‐0 surgical silk suture. The right carotid artery was isolated without ligation as a sham operation control. After surgery, the mice were nursed on a warming pad until they recovered.

### Histological Analysis of Atherosclerotic Lesions

Aortas were isolated from the mice (sparing as much as possible from the aortic root to the internal and external iliac arteries), and 4% paraformaldehyde was used to fix the aortas. Oil Red O (O0625; Sigma‐Aldrich) staining was applied to detect the total lesion area of the whole aorta. Hematoxylin and eosin staining was used to visualize the lesion area of the aortic root and carotid artery.

### Cells Culture

Human umbilical vein endothelial cells (HUVECs) were cultured in high‐glucose DMEM medium supplemented with 10% FBS and 1% penicillin–streptomycin under standard culture conditions (37 °C, 5% CO_2_). All HUVECs used for in vitro experiments were from passages up to 7.

### Shear Stress Experiments

For shear stress experiments, HUVECs were uniformly seeded onto polylysine‐coated glass slides and grown to a confluent monolayer under standard conditions. Shear stress was loaded onto HUVECs as previously described.^3^ In brief, the glass slides with monolayer HUVECs were assembled into rectangular parallel‐plate flow chambers (31–010; Glycotech) and then connected to the perfusion system. Peristaltic and syringe pump systems were used to apply LSS (12 ± 4 dyn/cm^2^) and OSS (0.5 ± 4 dyn/cm^2^) to the HUVECs. Cells on the glass slides that did not receive flow treatment served as static control. The cells flow systems were maintained at standard cell culture conditions. In some experiments, HUVECs were incubated with specific inhibitors or agonists for coculture before and during flow exposure.

### Lentivirus Infection

The short hairpin RNA lentiviral vectors targeting Piezo1, targeting YAP, and control vectors were purchased from Obio Technology (Shanghai, China). Lentivirus and 5 μg/mL polybrene were added to HUVECs for transduction. After 16 hours of lentivirus infection, the culture medium was removed and replaced with regular complete medium. Immunoblot analysis was used to assess the efficiency of silencing. HUVECs with Piezo1 silencing or YAP overexpressing were selected for subsequent experiments. The target information is shown in Tables S5 and S6.

### Intracellular Ca^2+^ Imaging

After treatment with LSS, OSS, or GsMTx4, Piezo1 agonist (Yoda1) HUVECs were loaded with Ca^2+^ fluorescent probes using Fluo‐4 AM (Beyotime, Shanghai, China) for 1 hour. Images of intracellular Ca^2+^ fluorescence intensity were acquired using an Olympus fluorescence microscope.

### Coimmunoprecipitation and Western Blot

Tissues and cells were sonicated and homogenized in RIPA Lysis buffers (with protease and phosphatase inhibitors) to extract protein samples. For immunoprecipitation, specific immunoprecipitation (FAK, Src) and IgG (immunoglobulin G) antibodies were used to bind protein overnight at 4 °C. The antibody–protein complex was adsorbed onto Protein A/G‐MagBeads and subsequently eluted in elution buffer. The obtained protein complex was subjected to subsequent Western blot analysis. Equal quantities of protein (10 mg) underwent separation via 8% to 10% SDS‐PAGE gel electrophoresis and were subsequently transferred to polyvinylidene fluoride membranes. Each membrane was incubated with 5% BSA at room temperature for 1 hour to achieve blocking. The membranes were immersed in dilutions containing primary antibodies and incubated overnight at 4 °C with slow shaking. Excess primary antibody was washed off with TBST, followed by incubation with the corresponding secondary antibody for 1 hour at room temperature. Ultrasensitive ECL chemiluminescent solution enabled the visualization of protein bands under a gel imaging system. Antibodies that had been bound to the membrane were eluted with the stripping buffer (CW0056M; CWBIO, China) and reincubated with another primary antibody to detect multiple proteins on a same membrane.

### Immunofluorescence Staining and Quantification

Fresh vascular tissue from mice were fixed in 4% paraformaldehyde for 24 hours and then embedded in paraffin. Tissue fragments (4 μm) were adhered to glass slides and subjected to deparaffinization, rehydration, and antigen repair, respectively. Cell samples were washed with prechilled PBS 3 times and then fixed in 4% paraformaldehyde for 20 minutes then permeabilized with 0.1% Triton X‐100 for 5 minutes (not needed for indicators expressed on the cell membrane). Samples were blocked with 5% donkey serum at room temperature for 1 hour, followed by overnight incubation with primary antibodies (in PBS) at 4 °C. After treating with fluorescein‐coupled secondary antibodies for 1 hour at room temperature in the dark, the samples were stained with DAPI for 10 minutes. Immunofluorescence images were obtained on an Olympus fluorescence microscope or a Leica SP8 stimulated emission depletion confocal fluorescence microscope.

Fluorescence images from the same experiment were obtained under the same microscope setup. For mouse experiments, quantification is based on analysis of 3 representative fluorescent images from 3 mice (1 fluorescent image for each mouse). Colocalization was calculated as follows: target protein (green) total fluorescence area/platelet‐endothelial cell adhesion molecule 1 (CD31) total fluorescence area×100%. For in vitro experiments, quantification is based on analysis of 1 fluorescent image per experiment (n=3–6). The average fluorescence intensity was obtained in ImageJ. For nuclear colocalization of yes‐associated protein was calculated as follows: nuclear fluorescence intensity/total cell fluorescence intensity×100%.

### Statistical Analysis

The statistical description and analysis of each part for this study are included in its corresponding figure and legend. Data analysis and statistical plots for this study were completed by GraphPad Prism software. Quantification was done from at least 3 independent experimental groups. Data from 2 groups were analyzed using the Student *t* test. Three or more groups underwent 1‐way ANOVA with Tukey post hoc test or 2‐way ANOVA with Bonferroni multiple comparison post hoc test. The results are displayed as mean±SEM, with statistical significance denoted in each figure.

## RESULTS

### Piezo1 Is Highly Expressed in Atherosclerotic Plaque and Disturbed Flow‐Exposed Endothelial Regions in ApoE−/− Mice

**Figure 1:**
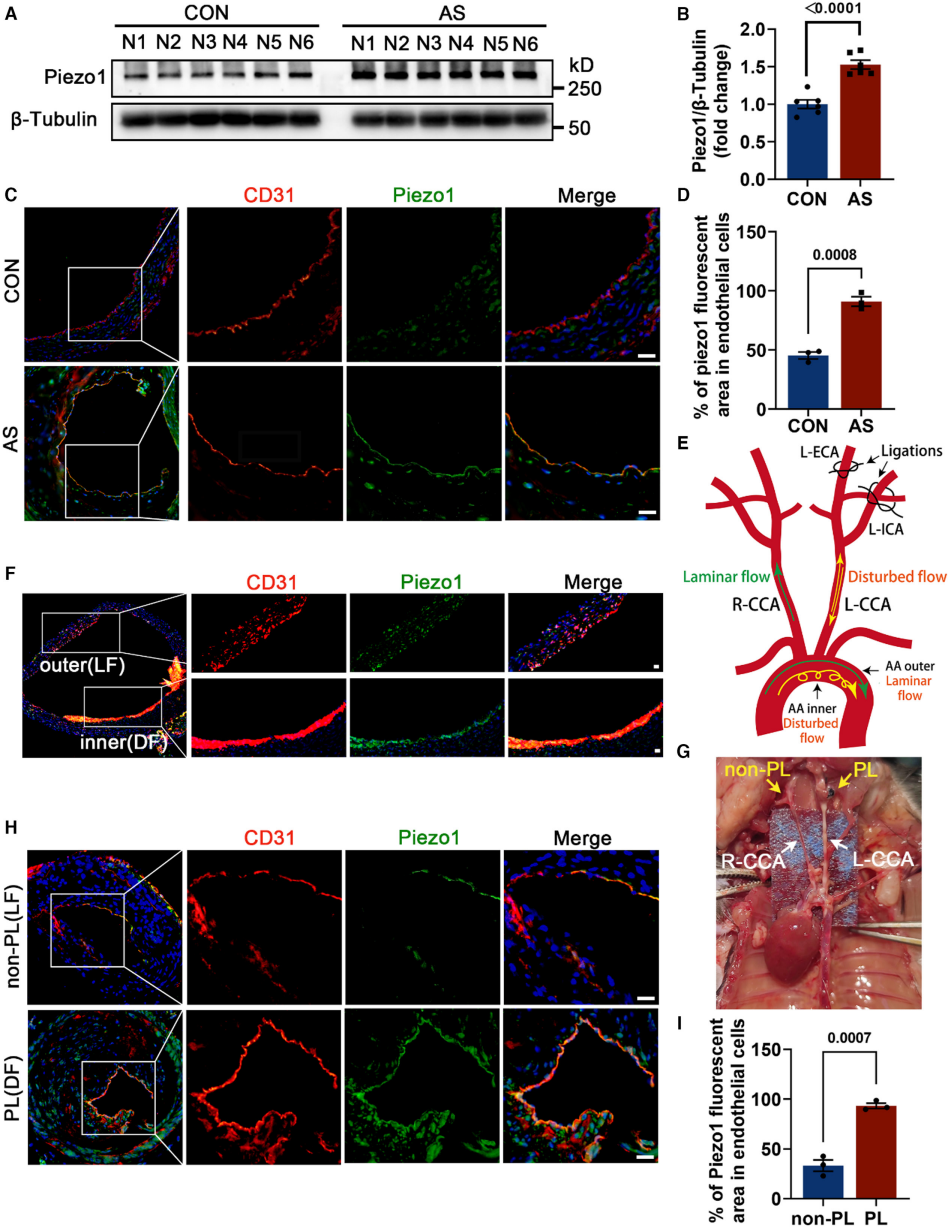
Piezo1 is highly expressed in atherosclerotic plaque and disturbed flow‐exposed endothelial regions in apoE−/− mice. **A** and **B**, Detection and quantification the protein expression level of Piezo1 in the aorta of apoE−/− mice from the CON and AS groups. **C** and **D**, Immunofluorescence images and analysis showed the expression and localization of Piezo1 in mice aortas from the CON and AS groups. Scale bar = 20 μm; n=3 mice per group. **E**, DF and LF patterns in atherosclerosis. **F**, Representative immunofluorescence images for Piezo1 in the outer and inner aortic arch. Scale bar = 20 μm. **G**, The anatomical diagram of partial carotid artery ligation. Ligation was performed at the left side, and the right side was the sham operation control. **H** and **I**, Immunofluorescence images and analysis for Piezo1 in the PL and non‐PL carotid arteries. Scale bar = 20 μm; n=3 mice per group. All data were statistically analyzed with unpaired 2‐tailed Student *t* test. ApoE indicates apolipoprotein E; AA, aortic arch; AS, atherosclerosis groups; CD31, platelet endothelial cell adhesion molecule 1; CON, control group; DF, disturbed flow; L‐CCA, left common carotid artery; L‐ECA, left external carotid artery; LF, laminar flow; L‐ICA, left internal carotid artery; N, 6 mice per group; PL, partial carotid artery ligation, and R‐CCA, right common carotid artery.

To begin with, we examined the expression of Piezo1 in the aortas of atherosclerotic apoE−/− mice fed a high‐fat Western diet. We revealed a significant upregulation of Piezo1 in atherosclerotic mice compared with those on a normal diet (Figures 1A and 1B). Immunofluorescence staining further showed that Piezo1 was upregulated and mainly colocalized with endothelial cells (labeled with CD31) in atherosclerotic lesions (Figures 1C and 1D). Given that Piezo1 is sensitive to flow shear stress, we next wondered whether the high expression of Piezo1 is associated with atherogenic DF. We examined the inner curvature of the aortic arch in apoE−/− mice, where the blood flow pattern is DF, and the outer curvature of the aortic arch, where the flow is a relative laminar flow (Figure 1E). Compared with the outer curvature of the aortic arch, the inner curvature of the aortic arch exhibited a higher level of Piezo1 (Figure 1F). To further confirm our findings, we performed partial carotid ligation in apoE−/− mice, a model that accelerates atherosclerosis through the local generation of DF and OSS^16^ (Figures 1E and 1G). ECs exposed to disturbed flow in the carotid arteries on the ligated side exhibited higher levels of Piezo1 compared to the non‐ligated laminar flow side (Figures 1H and 1I).

### Inhibition of Piezo1 Delays Long‐Term and Disturbed Flow‐Induced Atherosclerotic Plaque Progression and Endothelial Inflammation

**Figure 2:**
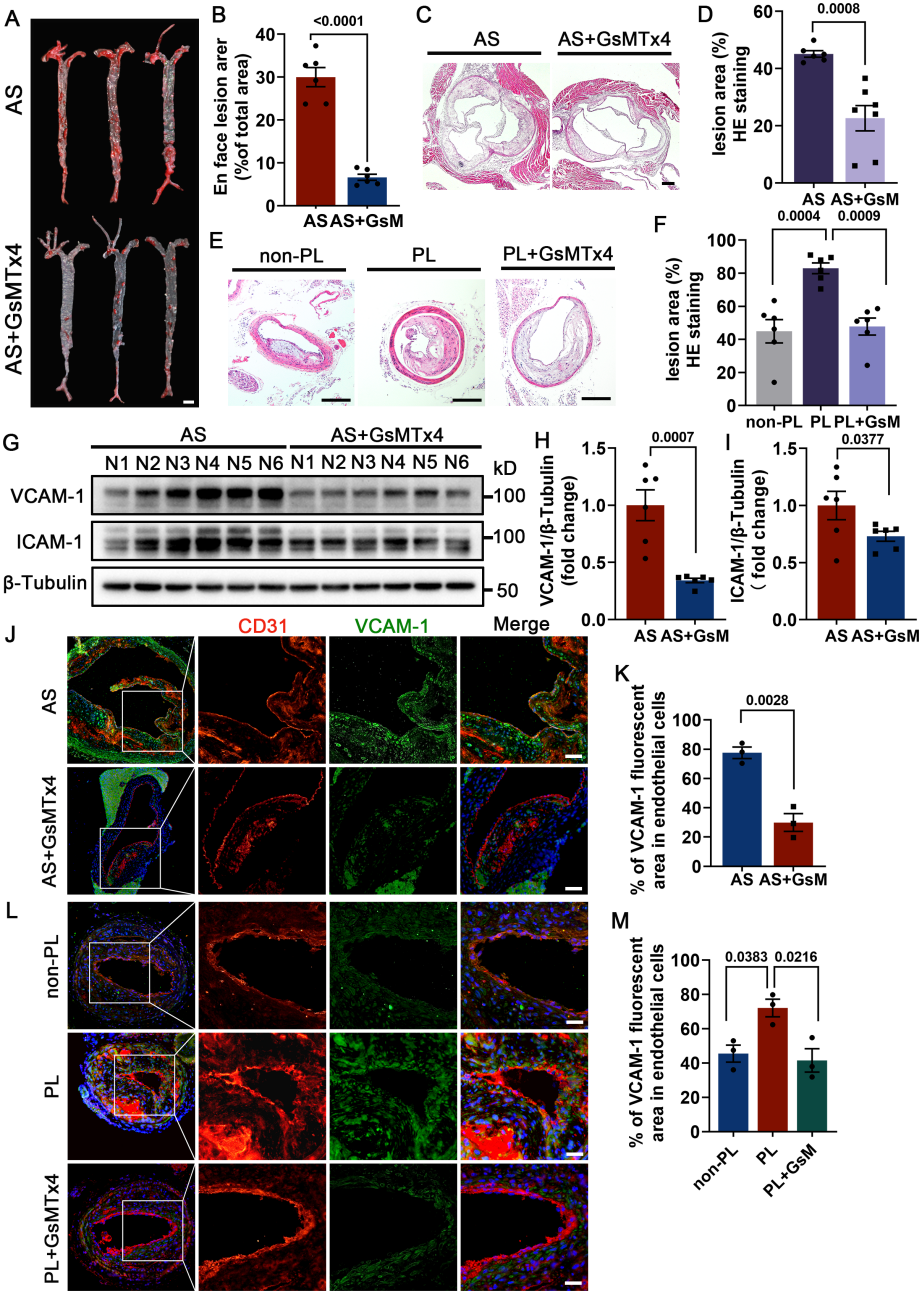
Inhibition of Piezo1 delays long‐term and disturbed flow‐induced atherosclerotic plaque progression and endothelial inflammation. **A**, En face Oil Red O staining of the whole aorta. **B**, Analysis and quantification the percentage of plaque area to total area; n=6 mice per group. **C** and **D**, Typical images of HE staining of the aortic root and quantification of the plaque area; n=6 mice per group. Scale bar = 200 μm. **E** and **F**, All mice were raised on a Western diet for 4 weeks. HE staining of carotid arteries and quantification of plaque area; n=6 mice per group. Scale bar = 200 μm. **G** through **I**, Western blot analysis and quantification for VCAM‐1 and ICAM‐1 in mice aortas; n=6 mice per group. **J** and **K**, ApoE−/− mice were injected with GsMTx4 or an equivalent dose of saline and maintained on a high‐fat diet for 12 weeks. **L** and **M**, PL‐apoE−/− mice were injected with GsMTx4 or an equivalent dose of saline and maintained on a high‐fat diet for 4 weeks. **J** and **K**, Immunofluorescence staining was conducted to evaluate the VCAM‐1 expression; n=3 mice per group. Scale bar = 20 μm. **B, D, H, I,** and **K**, Unpaired 2‐tailed Student *t* test. **F** and **M**, One‐way ANOVA with Tukey post hoc tests. ApoE−/− indicates apolipoprotein E; AS, atherosclerosis (Western diet fed) + saline (0.1 mg/kg per day) injection; AS+GsMTx4, atherosclerosis (Western diet fed) + GsMTx4 (0.1 mg/kg per day) injection; CD31, platelet endothelial cell adhesion molecule 1; GsM, GsMTx4; GsMTx4, Piezo1 inhibitor; HE, hematoxylin–eosin; ICAM‐1, intercellular adhesion molecule 1; PL, partial carotid ligation; PL + GsMTx4, partial carotid artery ligation+GsMTx4 (0.1 mg/kg per day) injection; and VCAM‐1, vascular cell adhesion molecule 1.

To explore Piezo1's function in atherosclerosis in vivo, apoE−/− mice were injected with GsMTx4, a selective pharmacological inhibitor of Piezo1, and were subjected to a high‐fat diet for 12 weeks. We evaluated the long‐term effect of Piezo1 on atherosclerosis. The inhibition efficiency of GsMTx4 on Piezo1 was detected by immunoblotting (Figures S1A and S1B). Oil red O staining and hematoxylin–eosin staining showed that the overall plaque burden and aortic root plaque area were significantly reduced in apoE−/− mice injected with GsMTx4 (Figure 2A through 2D). Next, we induced a DF atherosclerosis model by carotid artery ligation, in which we verified the role of Piezo1 in DF‐related atherosclerosis. Similarly, inhibition of Piezo1 notably reduced the progression of carotid plaque lesions associated with DF (Figures 2E and 2F). Furthermore, we explored Piezo1's role in endothelial inflammation, a critical step in the vascular response to DF during atherogenesis. Apparently, the inhibitor of Piezo1 (GsMTx4) attenuated the expression of atherosclerotic inflammatory markers VCAM‐1 and ICAM‐1 (Figure 2G through 2I). This finding was further confirmed by immunofluorescence staining (Figures 2J and 2K). In the partial carotid ligation model, DF‐induced VCAM‐1 overexpression was reversed by Piezo1 inhibition in the partial carotid ligation model (Figures 2L and 2M).

### Piezo1 Promotes Endothelial Inflammatory Activation in Response to OSS

**Figure 3:**
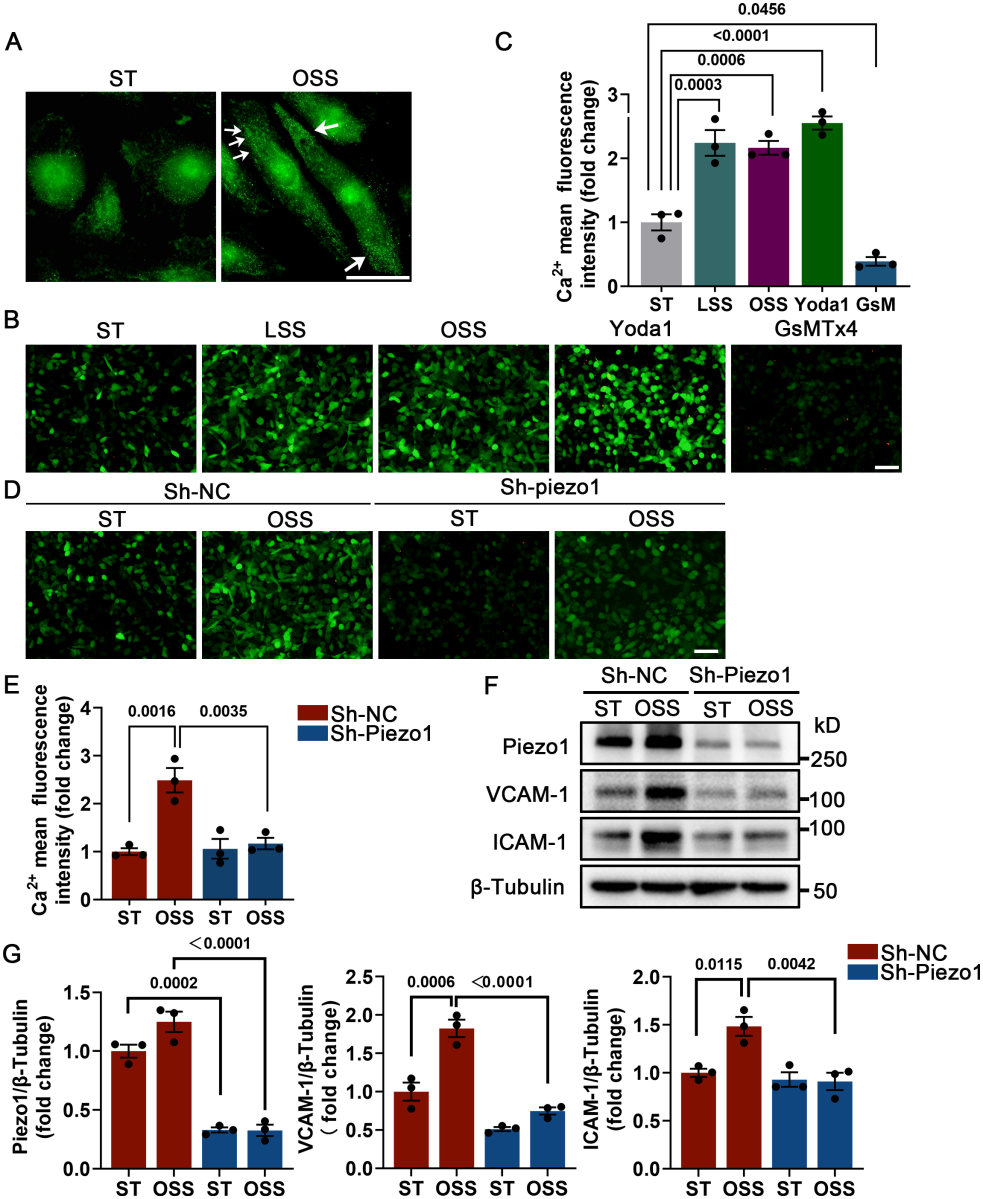
Piezo1 promotes endothelial inflammatory activation in response to oscillatory shear stress. **A**, Typical immunofluorescence staining image showing Piezo1 in HUVECs exposed to OSS (0.5 ± 4 dyn/cm^2^) or ST for 1 hour. Scale bar = 10 μm. **B** and **C**, HUVECs treated with Yoda1 (5 μm), GsMTx4 (5 μm), ST, and LSS (12 ± 4 dyn/cm^2^ or 0.5 ± 4 dyn/cm^2^) for 8 hours. Intracellular Ca^2+^ imaging and quantitative analysis. Scale bar = 50 μm. **D** and **E**, Imaging and quantification the Ca^2+^ fluorescence in sh‐NC and sh‐Piezo1 HUVECs after ST or OSS treatment for 8 hours. Scale bar = 50 μm. **F** and **G**, HUVECs from sh‐NC and sh‐Piezo1 groups were treated with ST or OSS for 8 hours, detection and quantification for VCAM‐1 and ICAM‐1. **C**, One‐way ANOVA with Tukey post hoc tests. **E** and **G**, Two‐way ANOVA with Bonferroni multiple comparison post hoc test. Pairwise comparisons were performed among the 4 groups. All experiments were independently repeated 3 times. Ca2+: calcium ion; GsM, GsMTx4; GsMTx4, Piezo1 inhibitor; HUVECs indicates human umbilical vein endothelial cells; ICAM‐1, intercellular adhesion molecule 1; LSS, laminar oscillatory shear stress; OSS, oscillatory shear stress; sh‐NC, lentivirus infection negative control; sh‐Piezo1, lentivirus target‐Piezo1 silencing; ST, static state; Yoda1: Piezo1 agonist; and VCAM‐1, vascular cell adhesion molecule 1.

To investigate how Piezo1 functions in ECs in response to DF, HUVECs in vitro were assembled into parallel‐plate flow chambers and loaded with OSS (0.5±4 dyn/cm^2^), mimicking DF stimulation acting on vascular intima. When remaining in a static state, Piezo1 was diffusely distributed in HUVECs, but when stimulated by OSS, local high‐density accumulation appeared (Figure 3A), which is consistent with a previous report.^10^ Considering the Ca^2+^ permeability properties of Piezo1, we examined Ca^2+^ influx to assess whether OSS could activate Piezo1. Intracellular Ca^2+^ imaging demonstrated that OSS stimulation promoted intracellular Ca^2+^ accumulation compared with a static state, which was consistent with the effect of the LSS and Piezo1 agonist Yoda1 but opposite to the inhibitor GsMTx4 (Figure 3B through 3C). Next, we performed Piezo1‐targeted silencing with lentiviral short hairpin RNA in HUVECs (Figure S2A and S2B). Piezo1 silencing significantly suppressed OSS‐mediated Ca^2+^ influx (Figure 3D through 3E). We later directed our attention to the involvement of Piezo1 in OSS‐induced endothelial inflammation. OSS increased mRNA level of endothelial inflammatory markers VCAM‐1 and ICAM‐1 (Figures S3A and S3B); the deficiency of Piezo1, however, partially canceled OSS‐induced VCAM‐1 and ICAM‐1 (Figure 3F through 3G).

### Piezo1 Promotes Endothelial Inflammation Through YAP Activation

**Figure 4:**
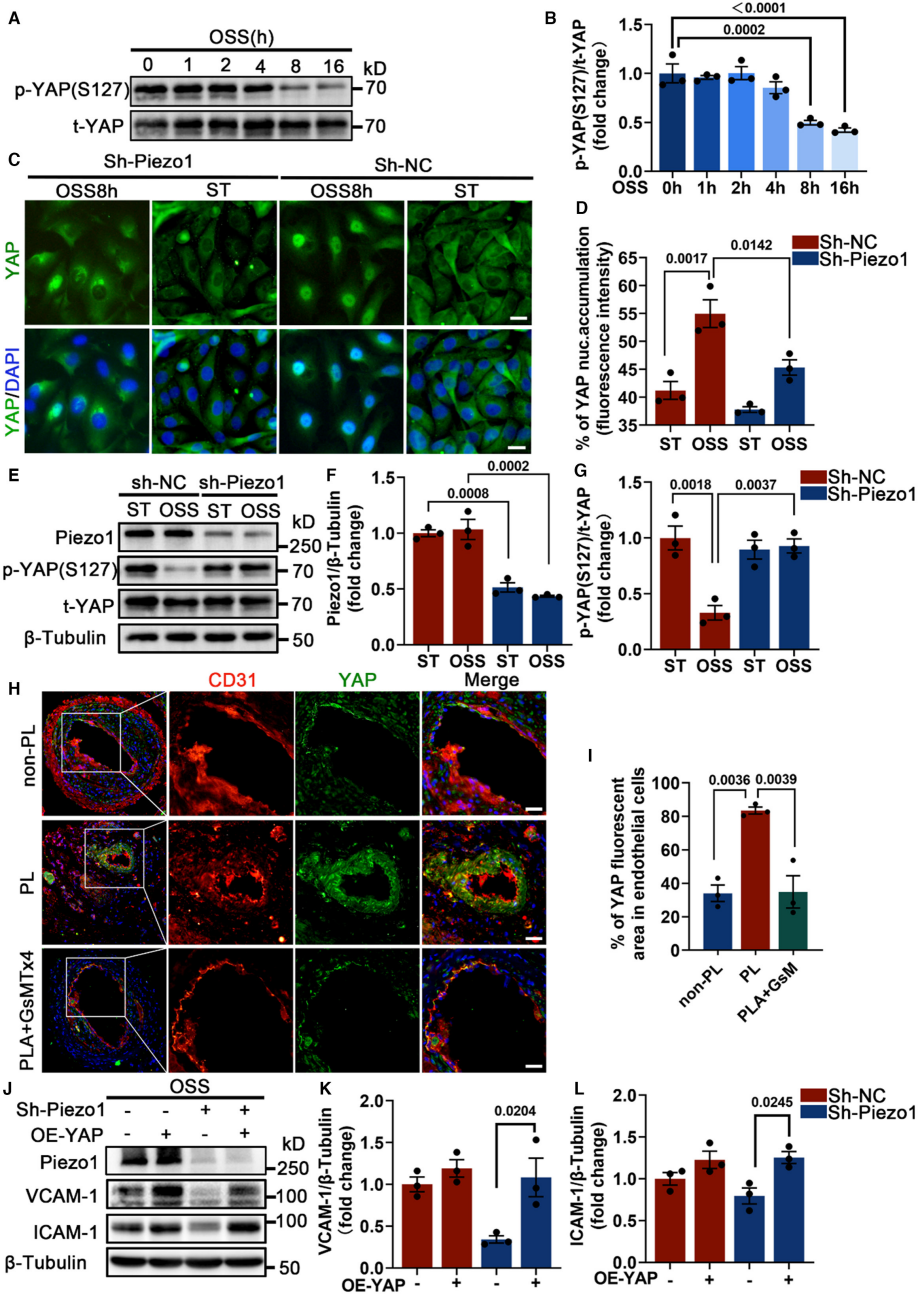
Piezo1 promotes endothelial inflammation through YAP activation. **A** and **B**, HUVECs were subjected to OSS for 0, 1, 2, 4, 6, 8, and 16 hours, respectively. Immunoblot analysis and quantification p‐YAP (S127) and t‐YAP. **C** and **D**, HUVECs with sh‐NC and sh‐Piezo1 were treated with ST or OSS for 8 hours. Immunofluorescence staining images displayed and analyzed for the nuclear accumulation of YAP. Scale bar = 20 μm. **E** through **G**, HUVECs (sh‐NC and sh‐Piezo1) exposed to ST or OSS for 8 hours. Immunoblot analysis and quantification for p‐YAP (S127)/t‐YAP. **H** and **I**, Immunofluorescence staining analysis and quantification for YAP in apoE−/− mice carotid arteries; n=3 mice per group. Scale bar = 20 μm. **J** through **L**, Western blot analysis demonstrated that silencing Piezo1 reversed the upregulation of VCAM‐1 and ICAM‐1 induced by YAP overexpression. **B** and **I**, One‐way ANOVA with Tukey post hoc tests. **D**, **F**, **G**, **K,** and **L**, Two‐way ANOVA with Bonferroni multiple comparison post hoc test. Pairwise comparisons among the 4 groups. All experiments were independently repeated 3 times. ApoE−/− indicates apolipoprotein E; HUVECs, human umbilical vein endothelial cells; CD31, platelet endothelial cell adhesion molecule 1; GsM, GsMTx4; GsMTx4, Piezo1 inhibitorPLA:partial left carotid artery ligation; ICAM‐1, intercellular adhesion molecule 1; nuc., nuclear; OE‐YAP, YAP overexpression; OSS, oscillatory shear stress; p‐YAP (S127), phosphorylation of YAP at Ser127; sh‐NC, lentivirus infection negative control; sh‐Piezo1, lentivirus target‐Piezo1 silencing; ST, static state; t‐YAP, total YAP; VCAM‐1, vascular cell adhesion molecule 1; and YAP, yes‐associated protein.

With the above findings in mind, we set out to investigate the potential connection between Piezo1 and endothelial inflammation. YAP has been reported to be an OSS‐sensitive transcription factor that can be activated by OSS to trigger endothelial inflammation and atherosclerosis.^17^, ^18^ Consistently, we found that OSS induced time‐dependent dephosphorylation of YAP at Serine 127 (Figures 4A and 4B). Immunofluorescence staining also showed strong YAP nuclear localization in HUVECs after exposed to OSS (Figures 4C and 4D). However, when Piezo1 was deleted, the OSS‐induced YAP activation was found to be partially inhibited, as shown by the inhibition of YAP dephosphorylation at Serine 127 and nuclear translocation (Figures 4C through 4G), proving that YAP was a significant downstream factor of Piezo1. Consistent with the in vitro results, we observed in vivo that partial carotid ligation‐induced DF resulted in higher YAP expression in the carotid endothelium compared with the nonpartial carotid ligation side, whereas the Piezo1 inhibitor GsTMx4 mitigated the upregulation of YAP (Figure 4H and 4I). We next performed rescue experiments and found that the downregulation of VCAM‐1 and ICAM‐1 mediated by Piezo1 silencing was reversed by YAP overexpression (Figure S4A through S4B; Figure 4J through 4L). Similarly, administration of inhibitor of large tumor suppressor kinase 1/2 (LATS1/2), a specific inhibitor of LATS1/2 (large tumor suppressor 1 and 2), the upstream inhibitory factor of YAP, was found to reverse the expression of VCAM‐1 (Figure S5A through S5D).

### Piezo1 Activates YAP Through Kinase FAK/Src

**Figure 5:**
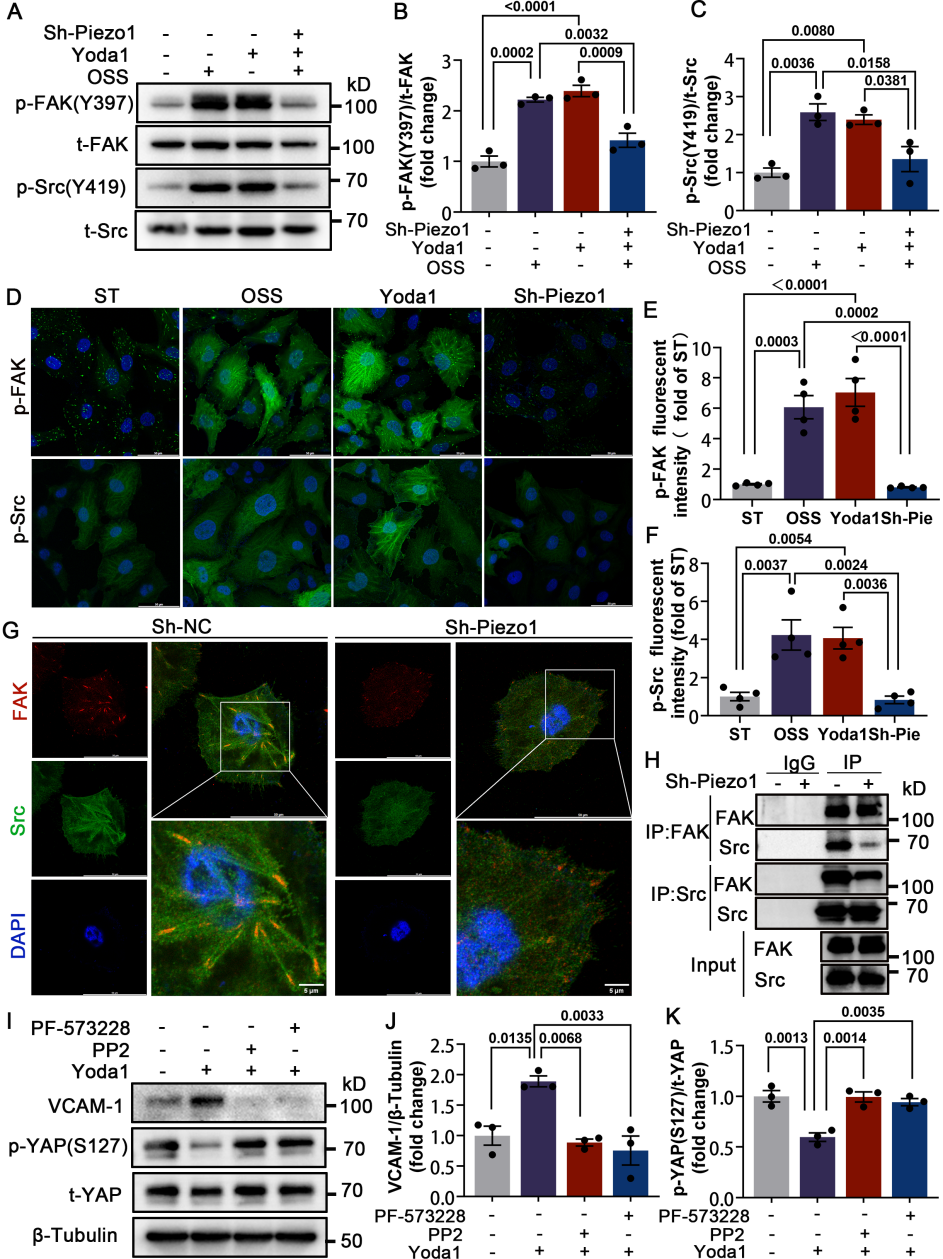
Piezo1 activates YAP through kinase FAK/Src. **A** through **F**, HUVECs of sh‐NC and sh‐Piezo1 were exposed to OSS or Yoda1 (5 μm) for 8 hours. Western blot (**A** through **C**) and immunofluorescence staining (**D** through **F**) analyzed and quantified the relative protein expression of p‐FAK (Y397) and p‐Src (Y419). Scale bar = 50 μm. **G**, Immunofluorescence staining examination of the spatial localization of FAK and Src in Piezo1‐silenced (sh‐Piezo1) and Piezo1‐normal (sh‐NC) HUVECs. Scale bars are shown in (**G**); n=4 independently repeated experiments. **H**, Coimmunoprecipitation analysis for the interaction of FAK and Src in Piezo1‐silenced (sh‐Piezo1) and Piezo1‐normal (sh‐NC) HUVECs. **I** through **K**, HUVECs were preincubated with Yoda1 (5 μm) followed by coincubation with PP2 (5 μm) or PF‐573228 (5 μm) for 24 hours. Western blot analyzed and quantified the relative expression of VCAM‐1 and p‐YAP (S127). All data were statistically analyzed with 1‐way ANOVA with Tukey post hoc tests. All experiments were independently repeated 3 times unless otherwise noted. FAK indicates focal adhesion kinase; p‐YAP (S127), phosphorylated YAP (Serine 127); p‐YAP, phosphorylated YAP; PF‐573228, FAK inhibitor; PP2, Src inhibitor; Src, proto‐oncogene tyrosine‐protein kinase; SRCt‐YAP, total YAP; Yoda1, Piezo1 agonist; HUVECs, human umbilical vein endothelial cells; IgG, immunoglobulin G; IP, immunoprecipitation; OSS, oscillatory shear stress; p‐FAK (Y397), phosphorylated FAK at Tyr397; p‐Src (Y419), phosphorylated Src at Tyr419; sh‐NC, lentivirus infection negative control; sh‐Piezo1, lentivirus target‐Piezo1 silencing; ST, static state; t‐FAK, total FAK; t‐Src, total Src; VCAM‐1, vascular cell adhesion molecule 1; and YAP, yes‐associated protein.

How Piezo1 transmits OSS mechanical signaling to YAP caught our attention. FAK and Src are important components for cellular mechanical signal sensing.^19^, ^20^ Previous studies have confirmed that Piezo1 activation modulates FAK activity^21^, ^22^; we thus investigated whether FAK/Src was involved in OSS‐mediated Piezo1 activation in HUVECs. Our findings indicated that the absence of Piezo1 reduced the levels of phosphorylation of FAK at Tyr397 (Tyrosine 397) (p‐FAK Y397) and Src at Tyr419 (Tyrosine 419) (p‐Src Y419) induced by OSS and Yoda1 (Figures 5A through 5F). Immunoprecipitation and immunofluorescence colocalization analysis showed that Piezo1 depletion decreased the coprecipitation and colocalization of FAK/Src (Figures 5G and 5H), suggesting that Piezo1 affects the binding of FAK/Src.

FAK and Src are known to activate YAP in mechanical reactions.^23^, ^24^, ^25^, ^26^ We thus theorized that inhibiting FAK and Src kinase activity may rescue Piezo1‐induced YAP activation and inflammation phenotype. In line with our hypothesis, treatment with the FAK inhibitor (PF‐573228, FAK inhibitor, 5 μm) and Src inhibitor (PP2, Src inhibitor 5 μm) (Figure S6A though S6D) reduced Yoda1‐induced YAP S127 dephosphorylation and VCAM‐1 expression (Figure 5I though 5K).

### Piezo1‐Mediated YAP Activation and Endothelial Inflammation Is Dependent on the Ca^2+^/CaM/CaMKII‐FAK/Src Axis

**Figure 6:**
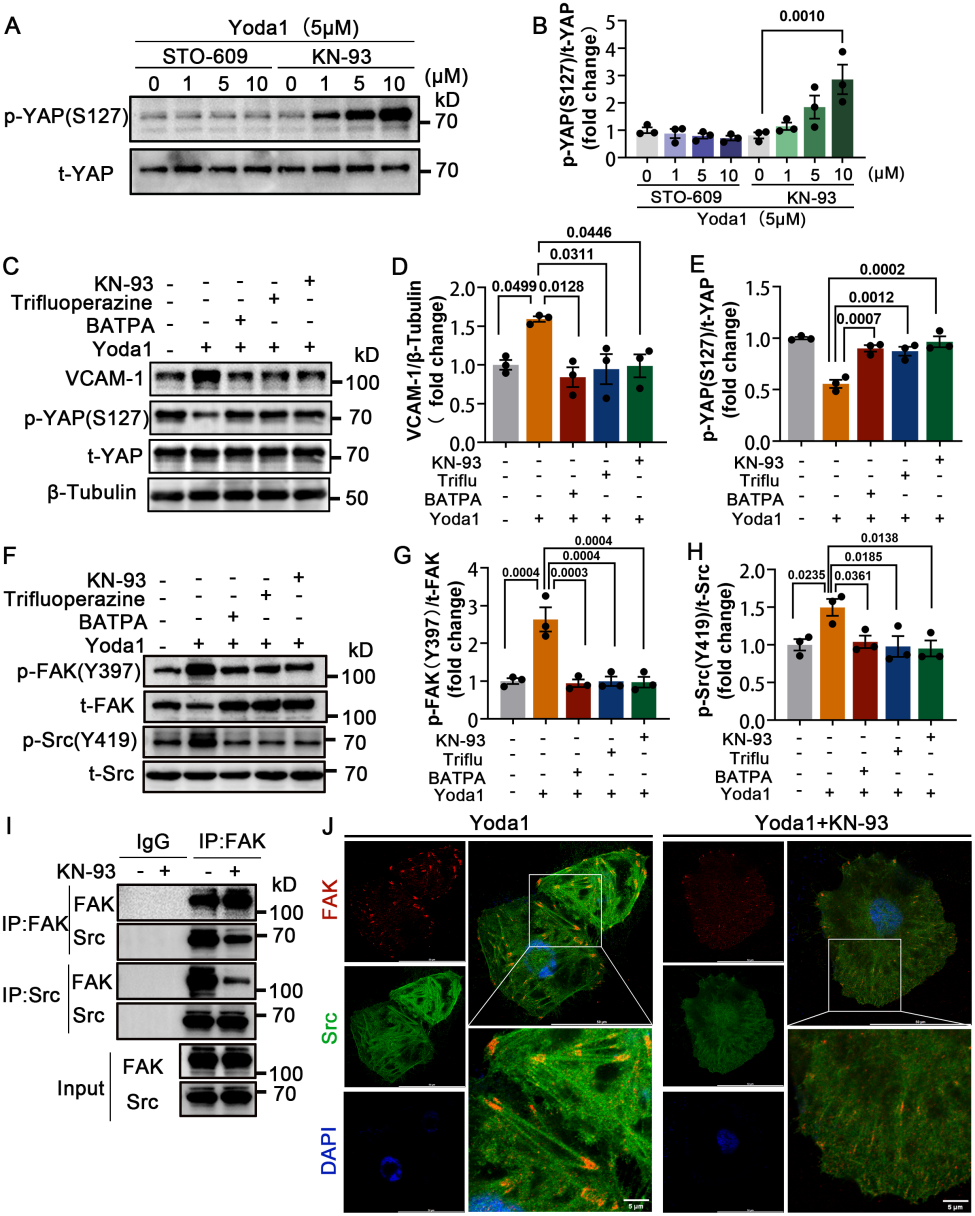
Piezo1‐mediated YAP activation and endothelial inflammation is dependent on Ca^2+^/CaM/CaMKII‐FAK/Src axis. **A** and **B**, HUVECs were preincubated with Yoda1 (5 μm) for 8 hours and followed by coincubation with STO‐609 (1, 5, 10 μM) or KN‐93 (1, 5, 10 μM) for 24 hours. Western blot analyzed and quantified the relative protein level of p‐YAP (S127). **C** through **E**, HUVECs were preincubated with Yoda1 (5 μm) for 8 hours and followed by coincubation, respectively, with BAPTA (10 μm), trifluoperazine (30 μm), and KN‐93 (10 μm) for 24 hours. Western blot analyzed and quantified the relative protein level of VCAM‐1 and p‐YAP (S127). **F** through **H**, HUVECs were incubated with BAPTA (10 μm), trifluoperazine (30 μm), or KN‐93 (10 μm) for 24 hours, respectively. Western blot analyzed and quantified the relative p‐FAK (Y397) and p‐Src (Tyr419). **I** and **J**, HUVECs treated with or without KN‐93 (10 μm) for 24 hours. **I**, Coimmunoprecipitation analysis for the interaction of FAK and Src. **J**, Immunofluorescence staining examination for the spatial localization of FAK and Src. Scale bars are shown in (**J**); n=4 independently repeated experiments. **B**, Two‐way ANOVA with Bonferroni multiple comparison post hoc test. **D**, **E**, **G,** and **H**, One‐way ANOVA with Tukey post hoc tests. All experiments were independently repeated 3 times unless otherwise noted. FAK indicates focal adhesion kinase; HUVECs, human umbilical vein endothelial cells; IP, immunoprecipitation; VCAM‐1, vascular cell adhesion molecule 1; and YAP, yes‐associated protein. Ca2+/CaM/CaMKs, calcium ions/calmodulin/calmodulin‐dependent protein kinases; STO‐609, CaMKK inhibitor; KN‐93, CaMKII inhibitor; p‐YAP (S127), phosphorylated YAP (Serine 127); Yoda1, Piezo1 agonist;BAPTA, Ca2+ chelator; p‐FAK Y397, phosphorylated FAK at Tyrosine 397; p‐Src(Y419), phosphorylated Src at Tyrosine 419; Src, proto‐oncogene tyrosine‐protein kinase SRC; t‐YAP, total YAP; t‐FAK, total FAK; p‐Src(Y419), phosphorylated Src at Tyrosine 419

Given that the activation of Piezo1 channels allows substantial Ca^2+^ influx, we guessed the classical calcium signaling pathway Ca^2+^/CaM/CaMKs (Ca^2+^/calmodulin/calmodulin‐dependent protein kinases) may be an important link between Piezo1 activation and its downstream events. Inhibitors of CaMKs (CaMKI, CaMKII, CaMKIV, and CaMKK) were used to assess which CaMKs works. STO‐609 CaMKK inhibitor (acts on CaMKK, including the substrates CaMKI and CaMKIV) and KN‐93 CaMKII inhibitor (works for CaMKII) were added separately to HUVECs. However, only the CaMKII inhibitor (KN‐93) canceled Piezo1‐mediated dephosphorylation of YAP S127 (Figure 6A and 6B). Next, we respectively treated HUVECs with Ca^2+^ chelator BAPTA Ca2+ chelator, CaM inhibitor trifluoperazine, and CaMKII inhibitor KN‐93. The results showed that Yoda1‐induced YAP dephosphorylation and VCAM‐1 expression were repressed by the Ca^2+^/CaM/CaMKII inhibitors (Figure 6C through 6E).

To further test if there was a connection between Ca^2+^/CaM/CaMKII and FAK/Src, HUVECs were treated with Ca^2+^, CaM, and CaMKII inhibitors, and the phosphorylation of FAK and Src was evaluated. We observed that the phosphorylation of FAK and Src were inhibited (Figure 6F though 6H). In addition, inhibition of CaMKII kinase activity resulted in reducing the interaction between FAK and Src (Figure 6I through 6J).

## DISCUSSION

**Figure 7:**
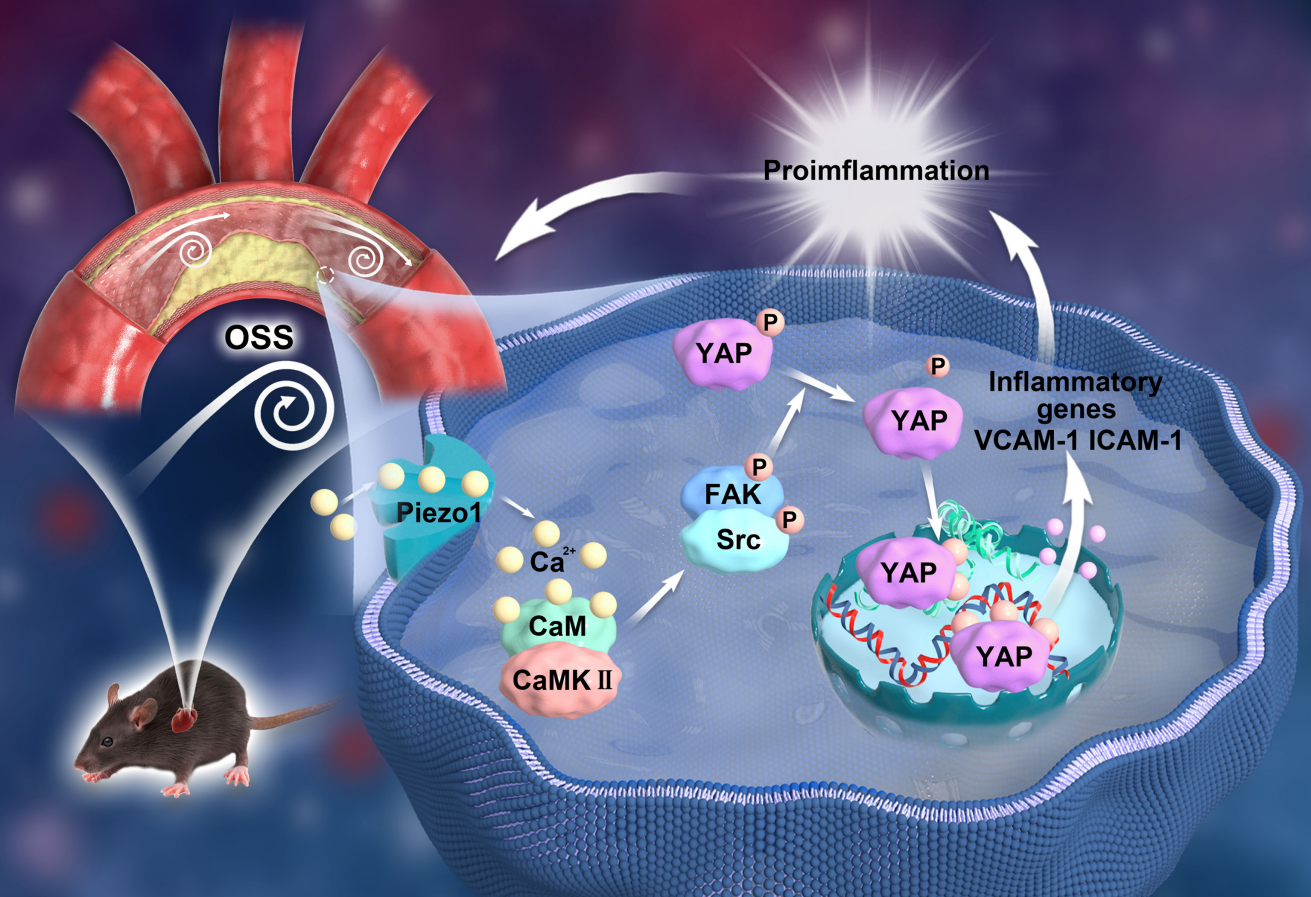
Schematic illustration of Piezo1 promotes atherosclerotic endothelial inflammation in response to OSS. The atherogenic DF/OSS activates endothelial Piezo1. The activation of Piezo1 allows Ca^2+^ influx, which subsequently activates the Ca^2+^/CaM/CaMKII signaling pathway and downstream kinases FAK/Src. This process contributes to YAP S127 dephosphorylation and ultimately triggers endothelial inflammation, promoting the progression of atherosclerosis. DF indicates disturbed flow; FAK, focal adhesion kinase; ICAM‐1, intercellular adhesion molecule 1; OSS, oscillatory shear stress; VCAM‐1, vascular cell adhesion molecule 1; and YAP, yes‐associated protein; Ca2+, calcium ions; Ca2+/CaM/CaMKs , calcium ions/calmodulin/calmodulin‐dependent protein kinases; Src, proto‐oncogene tyrosine‐protein kinase SRC; P, phosphorylation; YAP S127, YAP Serine12.

Although the mechanism by which OSS mediates inflammation activation in ECs and atherosclerosis remains an unsolved mystery, recent studies suggest that Piezo1, a mechanically sensitive protein, could be a key breakthrough.^21^, ^27^ Our observations revealed elevated Piezo1 expression in plaque tissue and ECs of apoE−/− atherosclerotic mice with chronic atherosclerosis and DF‐induced atherosclerosis. GsMTx4, a Piezo1 inhibitor, effectively decelerated plaque development and protected the endothelium against inflammation. OSS activated Piezo1 ECs and led to intracellular Ca^2+^ influx. Subsequently, it activated the Ca^2+^/CaMK/CaMKII signaling pathway and downstream kinases FAK/Src, resulting in YAP S127 dephosphorylation and nuclear translocation, ultimately triggering endothelial inflammation (Figure 7).

We initially observed the upregulation of Piezo1 in the plaque tissue and ECs of chronically atherosclerotic mice. Considering Piezo1's mechanosensitivity to blood flow shear stress, we observed high expression of Piezo1 in regions exposed to disturbed flow (DF), such as the inner aortic arch and the ligated carotid artery in atherosclerotic mice. Although the specific loss of endothelial Piezo1 reduced plaque burden in LDLR (low density lipoprotein receptor)−/− mice,^21^ global knockout of Piezo1 led to severe vascular developmental abnormalities and embryonic death.^10^, ^11^ Thus, we used the pharmacological inhibitor GsMTx4 to explore the in vivo role of Piezo1 on atherosclerosis. We found that GsMTx4 not only resists chronic atherosclerosis but also exhibits anti‐inflammatory and antiatherosclerotic effects in DF‐induced atherosclerosis, indicating that the atheroprotective role of Piezo1 may be associated with disturbed flow.

Previous research had already determined LSS activated Piezo1, leading to the accumulation of Piezo1 at the leading apical lamellipodia and Ca^2+^ influx,^6^, ^10^ thereby affecting the arrangement of endothelial cells.^10^ Piezo1 responded to both LSS and OSS as the same initial channel.^21^ We demonstrated that like LSS, OSS induced the local aggregations of Piezo1. As a nonselective cationic channel, one of Piezo1's immediate responses to shear stress is the facilitation of extracellular Ca^2+^ influx.^6^ We discovered that OSS also led to intracellular Ca^2+^ accumulation, which can be mimicked by Piezo1 agonist (Yoda1) and blocked by inhibitors (GsMTx4) or Piezo1 silence. Our data proved that OSS was sufficient to activate Piezo1.

Continuous OSS exposure leads to the activation of endothelial inflammatory signals and atherosclerosis development.^2^, ^3^, ^28^ In vivo, GsMTx4 reduced the upregulation of VCAM‐1 induced by DF from the inner curvature of the aortic arch and ligated carotid artery. GsMTx4 also had been reported to inhibit atherogenic inflammatory factors (JNK (c‐Jun N‐terminal kinase), TNF‐α (tumor necrosis factor alpha) and NF‐κB (nuclear factor‐kappaB)).^29^ Our data further demonstrated that in vitro, the OSS‐mediated upregulation of ICAM‐1 and VCAM‐1 were downregulated by the loss of Piezo1 in HUVECs. Additionally, the knock down of Piezo1 suppressed the OSS‐induced upregulation of NF‐kB and other inflammatory genes.^21^ Our evidence supported the critical role of Piezo1 in OSS‐induced atherosclerotic endothelial inflammation.

Our data further support that YAP serves as a key factor in Piezo1‐induced endothelial inflammation under OSS conditions. Piezo1 has been shown to activate the OSS‐sensitive factor YAP,^30^, ^31^, ^32^ but its specific mechanism remains unclear. The influx of Ca^2+^ is the main effect of Piezo1 channel opening, and intracellular accumulation of Ca^2+^ will trigger the calcium signaling pathway. FAK and Src are upstream kinases of YAP.^23^, ^33^, ^34^ We speculate that the calcium signal induced by Piezo1 may trigger downstream cascading kinases FAK/Src, thereby activating YAP. Here, we demonstrate that Piezo1‐induced Ca2+ triggers the Ca2+/CaM/CaMKII signal, mediating YAP dephosphorylation and nuclear translocation through FAK/Src kinases, thus triggering endothelial inflammation.

FAK is an important downstream kinase in Piezo1's response to shear stress.^21^, ^22^ The activation of Piezo1 by fluid shear stress resulted in downstream phosphorylation of Src.^35^, ^36^ In our study, we observed that the absence of Piezo1 reduced the OSS‐induced phosphorylation of FAK Y397 and Src Y419. More importantly, it decreased the formation of the FAK/Src complex. Tyrosine phosphorylation on FAK Y397 creates an SH2 (Src homology 2) binding site, recruiting Src and forming a dual‐activated FAK‐Src signaling complex.^37^ The assembly of FAK and Src kinase leads to phosphorylation of FAK and other tyrosine residues, maximizing kinase activity.^38^ We demonstrated that Piezo1‐dependent YAP activation requires FAK and Src kinases. The inhibition of FAK and Src hindered Piezo1‐mediated dephosphorylation of YAP S127 and upregulation of VCAM‐1. In intestinal epithelial inflammation, Src binds directly to YAP through its conserved SH3 (Src homology 3) domain and YAP SH3bm (Src homology 3 (SH3) binding motif) binding motif, activating YAP through Y357 phosphorylation.^39^ Whether Piezo1 affects the direct interaction between FAK/Src and YAP is worthy of further investigation.

Previous studies have emphasized the importance of calpain and calcineurin in the downstream targets of Piezo1‐Ca^2+^ regulation.^10^, ^31^, ^40^, ^41^ Here, we demonstrated the Ca2+/CaM/CaMKII pathway as an intermediate integrator between Piezo1 and YAP activation. We identified that CaMKII, rather than CaMKI or CaMKIV, reversed Piezo1‐mediated YAP S127 dephosphorylation. Previous reports of the association between Piezo1 and CaMKII partly supported our findings.^42^, ^43^, ^44^ There was a possibility that CaM directly bound to YAP and LATS1, forming a Ca^2+^‐dependent ternary complex, thereby facilitating Hippo signaling.^39^

Interestingly, we discovered that inhibitors of the Ca^2+^/CaM/CaMKII pathways reduced the phosphorylation levels of FAK and Src induced by Yoda1. The CaMKII inhibitor KN‐93 attenuated the coimmunoprecipitation and colocalization of FAK/Src. Our findings suggested that Piezo1‐triggered Ca^2+^/CaM/CaMKII signaling regulated the phosphorylation of FAK and Src, leading to the activation of YAP. In 3T3 cells (NIH 3T3 cells), blocking the Ca^2+^/CaM/CaMKII pathway and eliminating the rapid phosphorylation at the FAK Ser843 site. Activated CaMKII directly phosphorylates the recombinant carboxyl‐terminal region of FAK at the Serine843 residue.^45^ We speculated that the regulation of FAK/Src by CaMKII may occur through influencing the phosphorylation of tyrosine residues on FAK, thereby affecting the binding sites of FAK and Src. Further research is needed to substantiate this hypothesis.

Taken together, our research indicates that Piezo1 could be a potential therapeutic target for atherosclerosis. We elucidated the mechanotransduction role of Piezo1 in OSS‐mediated endothelial inflammation, identifying the Piezo1‐Ca^2+^/CaM/CaMKII‐FAK/Src‐YAP axis as a novel signaling cascade. However, it is uncertain whether other factors, such as membrane receptor crosstalk, interaction between adaptor proteins and kinases, and synergistic or inhibitory effects of other transcription factors, are also involved in the Piezo1‐mediated mechanotransduction pathway. The construction of a more complete endothelial mechanotransduction network is worth further exploration in the future.

## Sources of Funding

This research was supported by the Joint Project on Regional High‐Incidence Diseases Research of Guangxi Natural Science Foundation (grant number 2023GXNSFAA026135), National Natural Science Foundation of China (grant numbers 82060226, 82360248), and the Innovation Project of Guangxi Graduate Education (grant number YCBZ2024137).

## Disclosures

None.

## Supporting information

Tables S1–S6
Figures S1–S6
Data S1
