# Supplementary material for: Piezo1‐Mediated Mechanotransduction Contributes to Disturbed Flow‐Induced Atherosclerotic Endothelial Inflammation
Source: J Am Heart Assoc. 2024 Oct 25;13(21):e035558. doi: 10.1161/JAHA.123.035558 (PMC11935715; doi:10.1161/JAHA.123.035558)

SUPPLEMENTAL MATERIAL

Table S1. Animals (in vivo studies).

Species	Vendor or Source	Background	Sex	Persistent ID / URL
Wide type mice	Nanjing Junke Bioengineering Corporation, Ltd. (NanJing,China)	C57BL/6J	Male	NA
ApoE-/- mice	Nanjing Junke Bioengineering Corporation, Ltd. (NanJing,China)	C57BL/6J	Male	NA

Table S2. Cultured Cells.

Name	Vendor or Source	Sex (F, M, or unknown)	Persistent ID / URL
HUVECs cell line	Procell	Unknown	NA

Table S3. Major antibodies.

Target	Source	Catalog #	Working concentration	Persistent ID / URL
Piezo1	Proteintech	15939-1-AP	WB (Western Blot), 1:500 IF(Immunofluorescence), 1:50	https://www.ptgcn.com/products/FAM38A-Antibody-15939-1-AP.htm
Piezo1	Proteintech	28511-1-AP	WB, 1:500	https://www.ptgcn.com/products/PIEZO1-Antibody-28511-1-AP.htm
CD31	Servicebio	GB12064	IF, 1:500	https://www.servicebio.cn/goodsdetail?id=4868
VCAM-1	Abcam	ab134047	WB, 1:5000 IF, 1:100	https://www.abcam.cn/products/primary-antibodies/vcam1-antibody-epr5047-ab134047.html
VCAM-1	Santa Cruz	Sc-13160	WB, 1:500	https://www.scbt.com/zh/p/vcam-1-antibody-e-10?requestFrom=search
ICAM-1	Proteintech	10831-1-AP	WB, 1:2000 IF, 1:100	https://www.ptgcn.com/products/ICAM-1-Antibody-10831-1-AP.htm
ICAM-1	Santa Cruz	sc-8439	WB, 1:500	https://www.scbt.com/zh/p/icam-1-antibody-g-5?requestFrom=search
YAP	Cell Signaling Technology	14074	WB, 1:1000 IF, 1:100	https://www.cellsignal.cn/products/primary-antibodies/yap-d8h1x-xp-rabbit-mab/14074?site-search-type=Products&N=4294956287&Ntt=yap&fromPage=plp
YAP	Proteintech	13584-1-	WB, 1:5000	https://www.ptgcn.com/products/Y

		AP		AP1-Antibody-13584-1-AP.htm
FAK	Proteintech	66258-1-Ig	WB, 1:5000<br>IF, 1:400	https://www.ptgcn.com/products/FAK-Antibody-66258-1-Ig.htm
FAK	Cell Signaling Technology	3285	IP(Immunoprecipitation), 1:50	https://www.cellsignal.cn/products/primary-antibodies/fak-antibody/3285
Src	Zen Bioscience	320001	WB, 1:1000	http://www.zen-bio.cn/prod_view.aspx?IsActiveTarget=True&TypeId=180&Id=553291&FId=t3:180:3
Src	Cell Signaling Technology	2109	IP, 1:50<br>WB, 1:1000<br>IF, 1:100	https://www.cellsignal.cn/products/primary-antibodies/src-36d10-rabbit-mab/2109
YAP (phospho S127)	Abcam	Ab76252	WB, 1:1000	https://www.abcam.cn/products/primary-antibodies/yap1-phospho-s127-antibody-ep1675y-ab76252.html
p-FAK Y397	Abmart	T55587S	WB, 1:2000	http://www.ab-mart.com.cn/page.aspx?node=%2077%20&id=%201827
p-Src Tyr419	Affinity	AF3162	WB, 1:1000	https://www.affbiotech.cn/goods-1343-AF3162-Phospho_Src_Tyr419_Antibody.html
β-tubulin	Abmart	M20005	WB, 1:5000	http://www.ab-mart.com.cn/page.aspx?node=%2059%20&id=%20983
anti-Mouse Secondary Antibody HRP conjugated	Signalway antibody	L3032	WB, 1:5000	https://www.sabbiotech.cn/g-3810-Goat-anti-Mouse-IgG-Secondary-AntibodyHRP-conjugated-L3032.html
anti-Rabbit Secondary Antibody HRP conjugated	Signalway antibody	L3012	WB, 1:5000	https://www.sabbiotech.cn/g-3806-Goat-anti-Rabbit-IgG-Secondary-AntibodyHRP-conjugated-L3012.html
Cy3 conjugated Anti-Mouse	Servicebio	GB21401	IF, 1:500	https://www.servicebio.cn/goodsdetail?id=254
anti-Rabbit Secondary Antibody, FITC	Thermo Fisher Science	F2765	IF, 1:500	https://www.thermofisher.cn/cn/zh/antibody/product/Goat-anti-Rabbit-IgG-H-L-Cross-Adsorbed-Secondary-Antibody-Polyclonal/F-2765

Table S4. Inhibitors.

**Description**	**Source /**	**Persistent ID / URL**

	Repository	
GsMTx4	MedChemExpres (HY-P1410)	https://www.medchemexpress.cn/gsmtx4.html
Yoda1	MedChemExpress (HY-18723)	https://www.medchemexpress.cn/GlyT2-IN-1.html
GA-017	MedChemExpress (HY-147082)	https://www.medchemexpress.cn/ga-017.html
BATPA	MedChemExpress (HY-100168）	https://www.medchemexpress.cn/BAPTA.html
Trifluoperazine	MedChemExpress (HY-B0532）	https://www.medchemexpress.cn/trifluoperazine.html
KN-62	MedChemExpress （HY-13290）	https://www.medchemexpress.cn/KN-62.html
PP2	MedChemExpress (HY-13805）	https://www.medchemexpress.cn/PP2.html
PF-573228	MedChemExpress （HY-10461）	https://www.medchemexpress.cn/PF-573228.html

Table S5. PCR primers used for RT-qPCR in the study.

Gene	Forward (5'-3')	Reverse (5'-3')
GAPDH homo	GCATCCTGGGCTACACTGA	CCAGCGTCAAAGGTGGAG
ICAM-1 homo	GGATAATGTTTGCAGCTTCTCA	ATTCGTCACCTTCCCATTCAGT
VCAM-1 homo	AGTGACCATCTACAGCTTTCCG	CTTCACTGTCACCTCGGTCC

Table S6. siRNA sequence used for lentivirus infection in the study.

Target	Target Sequence (5'-3')
Piezo1 #1	CTCACCAAGAAGTACAATCAT
Piezo1 #2	GCTGCTCTGCTACTTCATCAT
Piezo1 #3	GTACAACGTCACCGTCATCAT
NC	CCTAAGGTTAAGTCGCCCTCG

Figure S1. GsMTx4 inhibits Piezo1 protein expression in apolipoprotein E knockout (ApoE −/−) mice aortas.

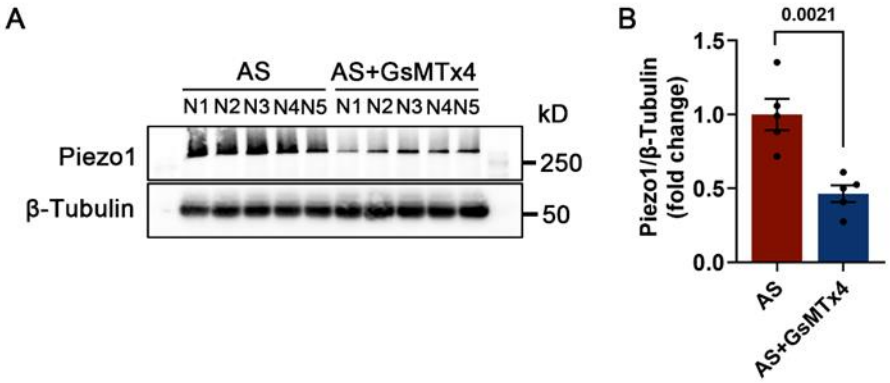

(**A**) Western blot analysis for aortas protein lysate from ApoE-/- mice after fed high-fat diet for 12 weeks, n=5 each group. (**B**) Quantitative analysis of Piezo1 protein expression in aortas. Unpaired 2-tailed Student t test, n=5 each group. AS=atherosclerosis (western diet fed) +saline(0.1mg/kg/day) injection, AS+GsMTx4=atherosclerosis (western diet fed) +GsMTx4(0.1mg/kg/day) injection.

Figure S2. Validation of the shRNA-mediated silence efficiency of Piezo1 in human umbilical vein endothelial cells.

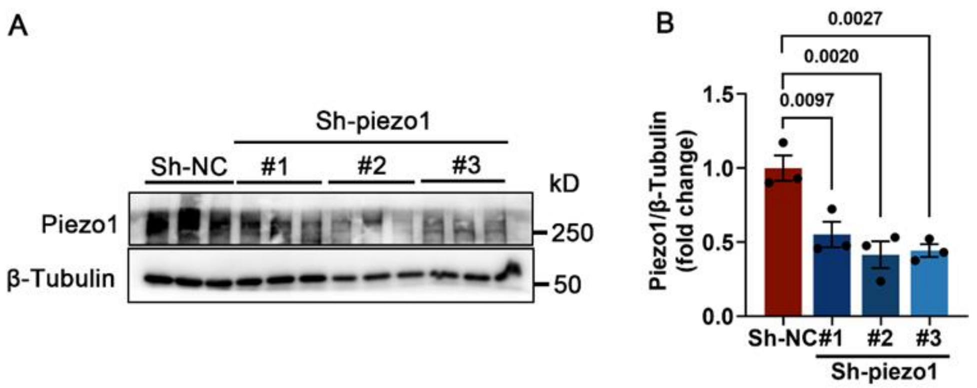

(**A**) HUVECs were infected with lentiviruses containing Piezo1-targeted (Sh-Piezo1) or control (Sh-NC) shRNA. Three interference targets (sh-Piezo1#1、#2 and#3) were designed, as listed in Table S2. The silence efficiency was assessed by western blot analysis. (**B**) Quantization of relative Piezo1 protein expression. Statistical significance was assessed by one-way ANOVA with Tukey's post-hoc tests, n=3 each group. Sh-NC=lentivirus infection negative control, Sh-Piezo1=lentivirus target-Piezo1 silencing.

Figure S3. Oscillatory shear stress (OSS) promotes the expression of VCAM-1 and ICAM-1 in human umbilical vein endothelial cells.

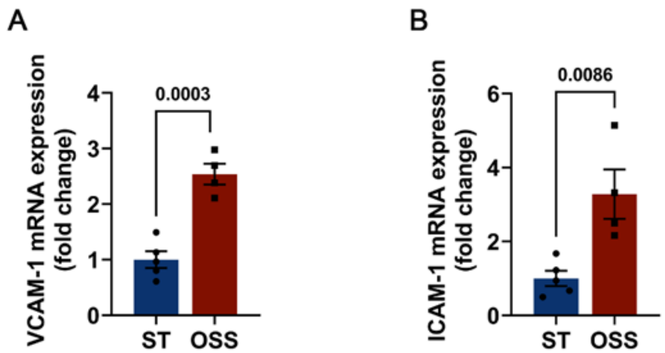

(**A**)HUVECs were exposed to ST or OSS for 8 hours, the expression of VCAM-1 and ICAM-1 was assessed by RT-qPCR using GAPDH as a housekeeping gene. ST= Resting state; OSS= Oscillatory shear stress. (**B**) Quantization of VCAM-1(A) and ICAM-1(B) relative mRNA expression. Statistical significance was assessed by unpaired 2-tailed Student t test, n=5 for ST group and n=4 for OSS group. ST=static condition, OSS=oscillatory shear stress.

Figure S4. Overexpression efficiency of YAP in human umbilical vein endothelial cells.

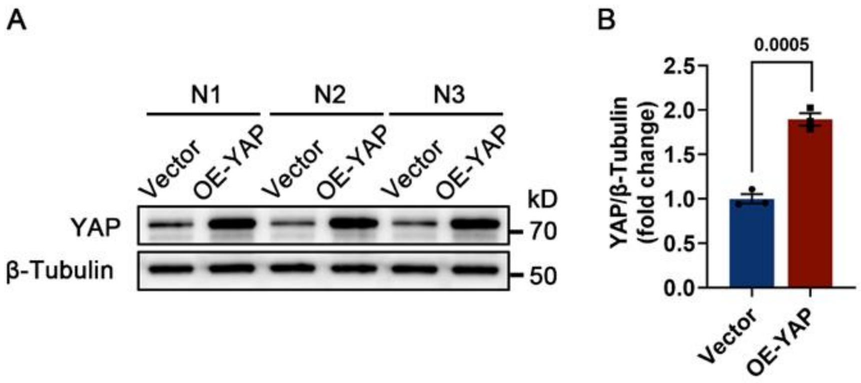

(**A**) HUVECs were infected with lentiviruses containing YAP-targeted (OE-YAP) or control (Vector) shRNA. Overexpression efficiency of YAP detected by Western blot. (**B**) Quantization of relative YAP protein expression. Statistical significance was assessed by Student's t-test, n=3 each group. OE-YAP=YAP overexpression

Figure S5. Deletion of Piezo1 reverses YAP-mediated upregulation of VCAM-1.

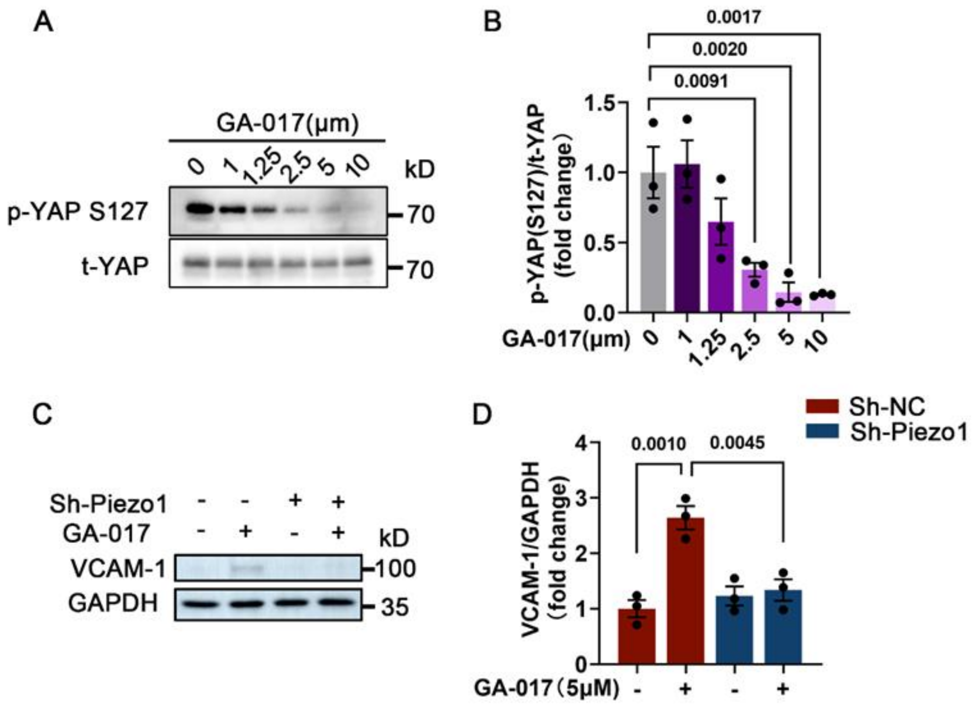

(**A**) HUVECs were treated with GA-017（0, 1, 1.25, 5, 10 μm）for 24 hours, western blot was used to detect YAP S127. (**B**) Analysis and quantification of p-YAP S127; one-way ANOVA with Tukey's post-hoc tests, n=3 each group. (**C**) Western blot analysis shows that the absence of Piezo1 reverses the effect of GA-017 on VCAM-1. (**D**) Analysis and quantification for VCAM-1. Statistical significance was assessed by two-way ANOVA with Bonferroni multiple comparison post-hoc test, n=3 each group. P-YAP (S127) =phosphorylation of YAP at Ser127; t-YAP=total YAP.

Figure S6. Inhibition efficiency of FAK inhibitor (PF-573228) and Src inhibitor (PP2).

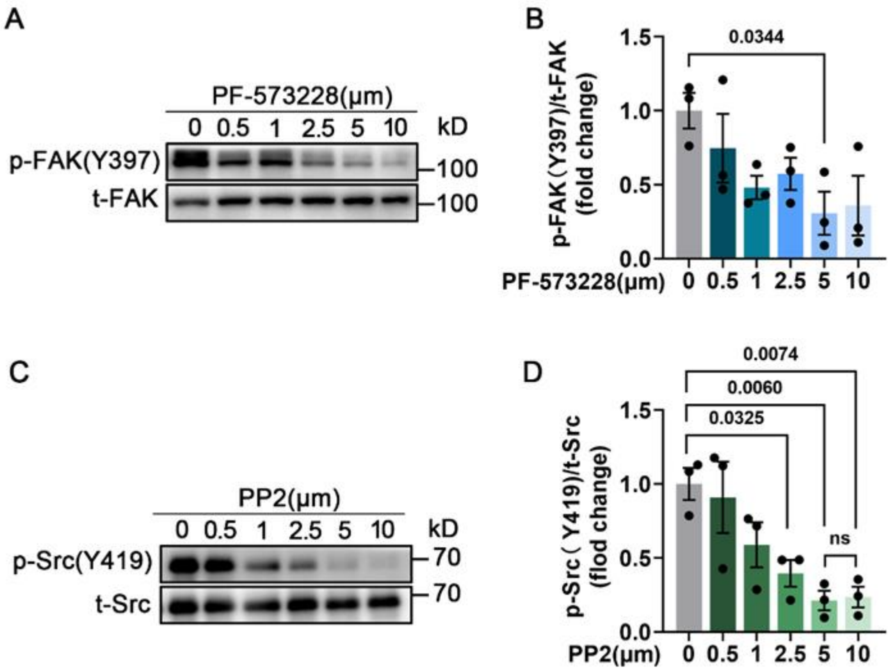

(**A**) HUVECs were treated with FAK inhibitor PF-573228(0,0.5,1,2.5,5,10 μ m) and (**C**) with Src inhibitors PP2(0,0.5,1,2.5,5,10 μ m) for 24 hours. The inhibitory efficiency was assessed by western blot analysis. Analysis and quantification for (**B**) phosphorylated FAK at Tyr127 (p-FAK Y397) and (**D**) phosphorylated Src at Tyr416 (p-Src Tyr416). Statistical significance was assessed by one-way ANOVA with Tukey's post-hoc tests, n=3 each group. P-FAK (Y397) = phosphorylated FAK at Tyr 397, t-FAK=total FAK, p-Src (Y419) = phosphorylated Src at Tyr419, t-Src=total Src, IP=immunoprecipitation.

Uncropped Gels

Full uncropped gels for Figure 1A

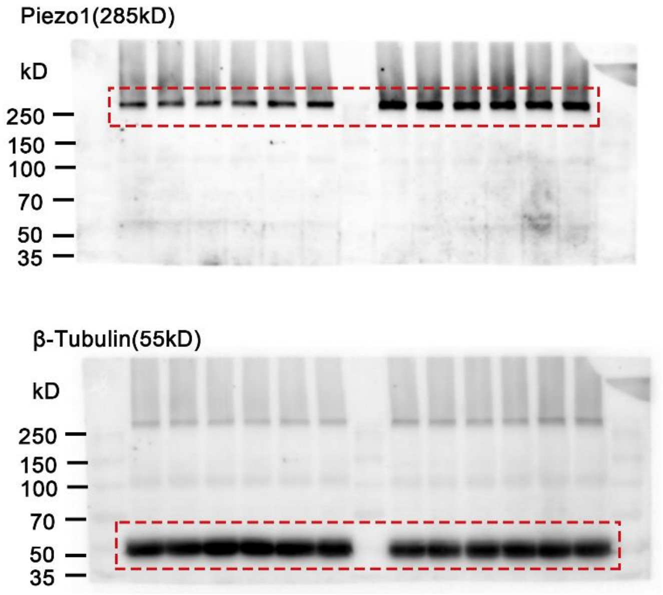

Full uncropped gels for Figure 2G

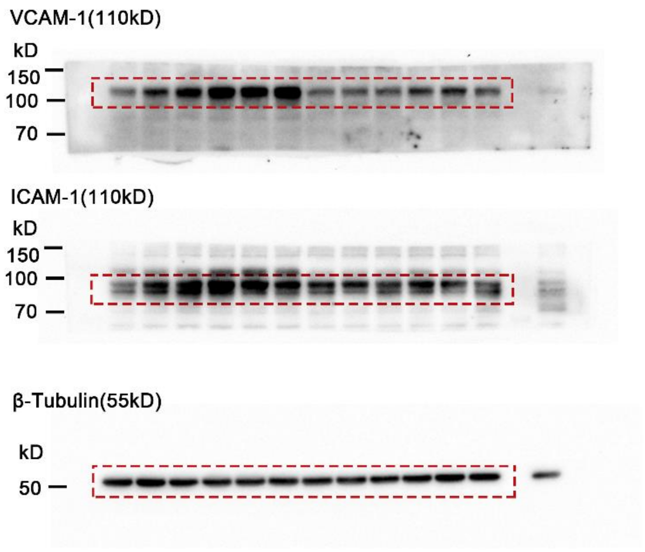

Full uncropped gels for Figure 3F

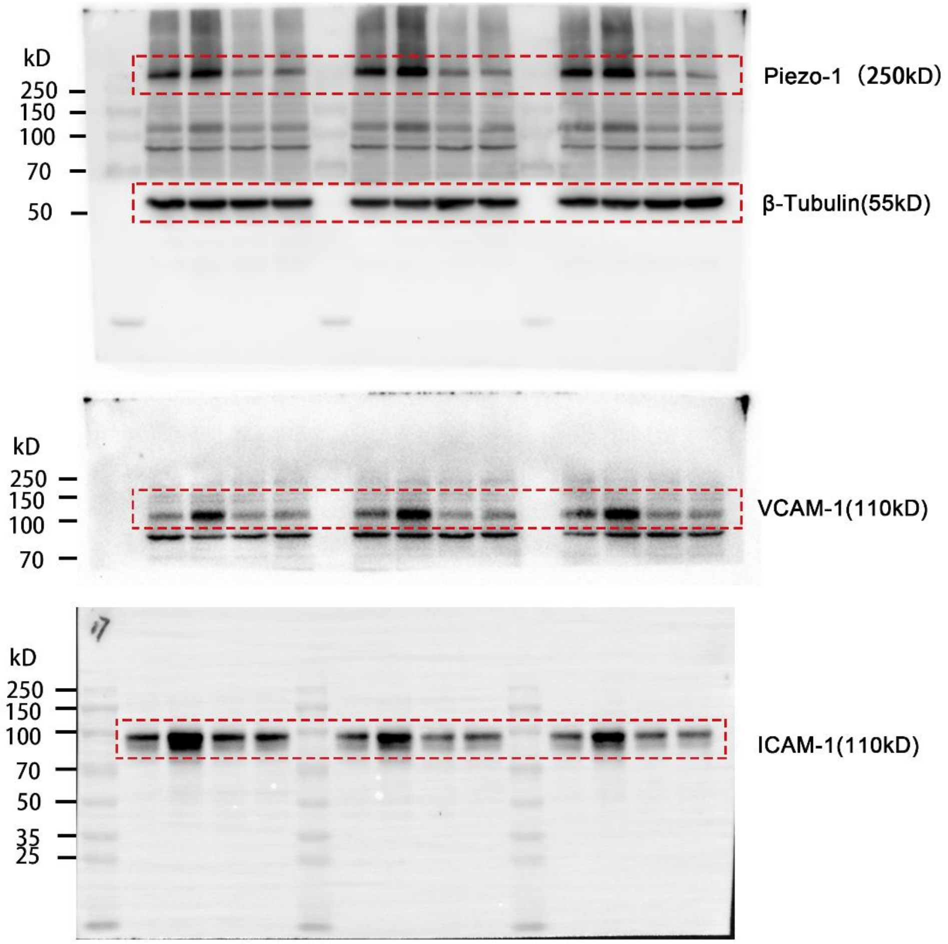

Full uncropped gels for Figure 4A

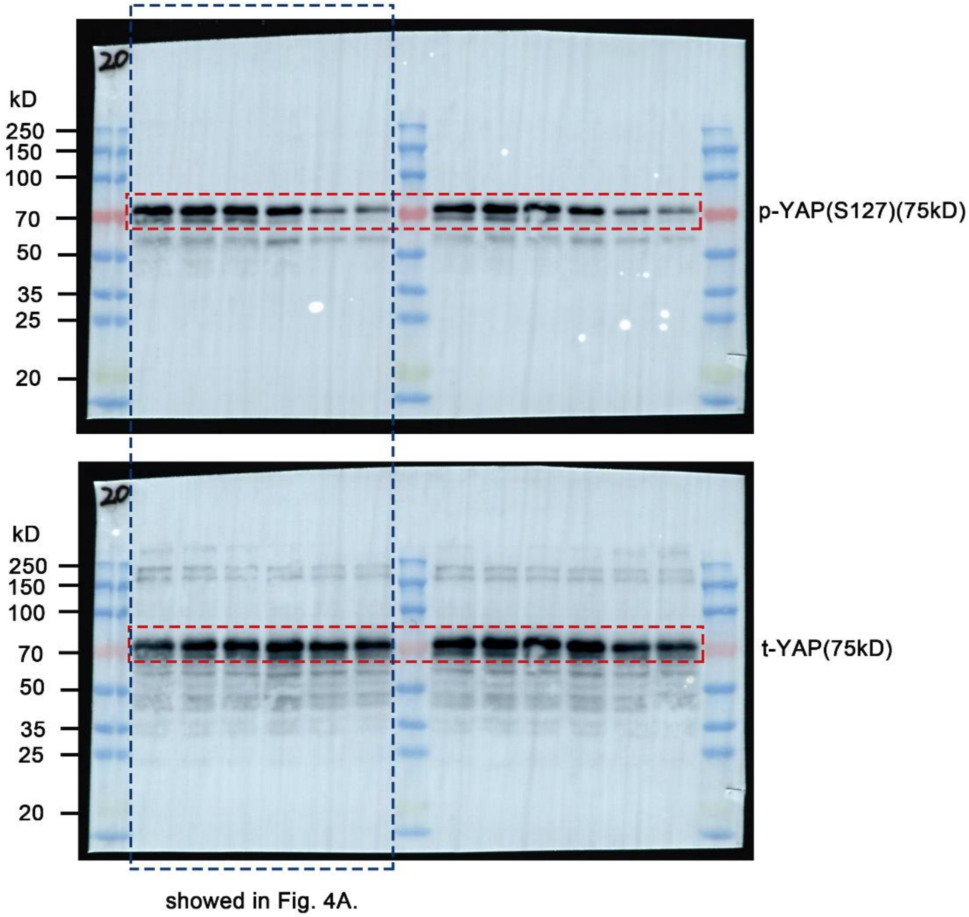

Full uncropped gels for Figure 4E

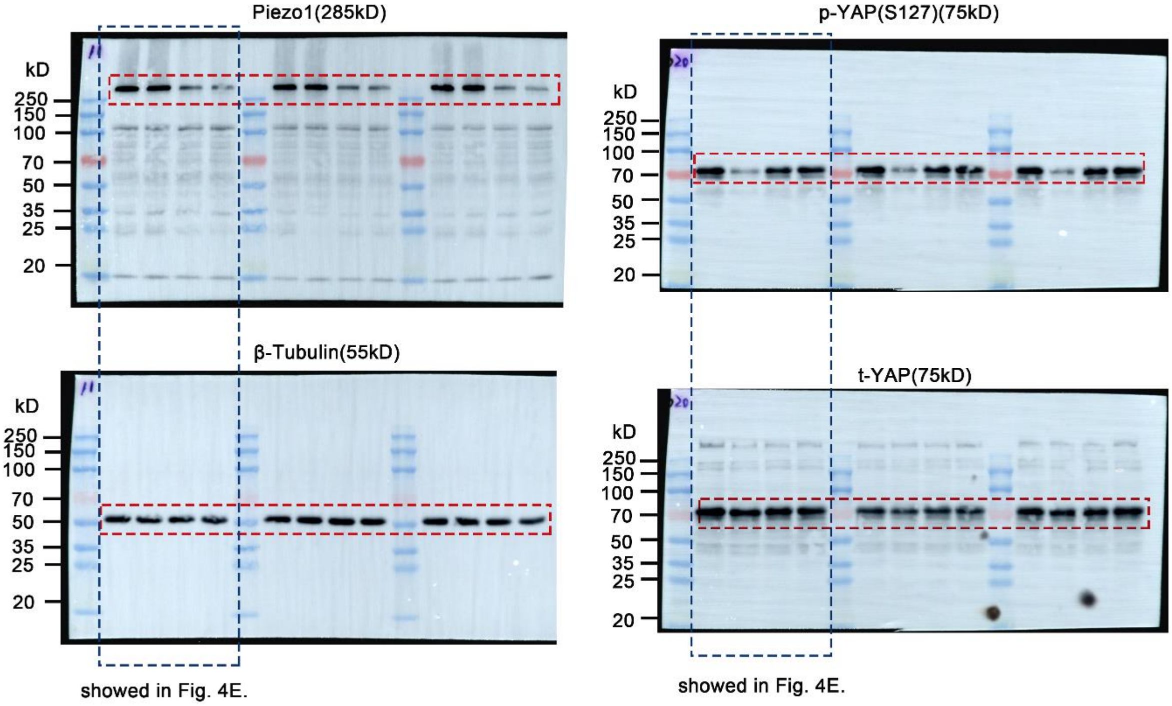

Full uncropped gels for Figure 4J

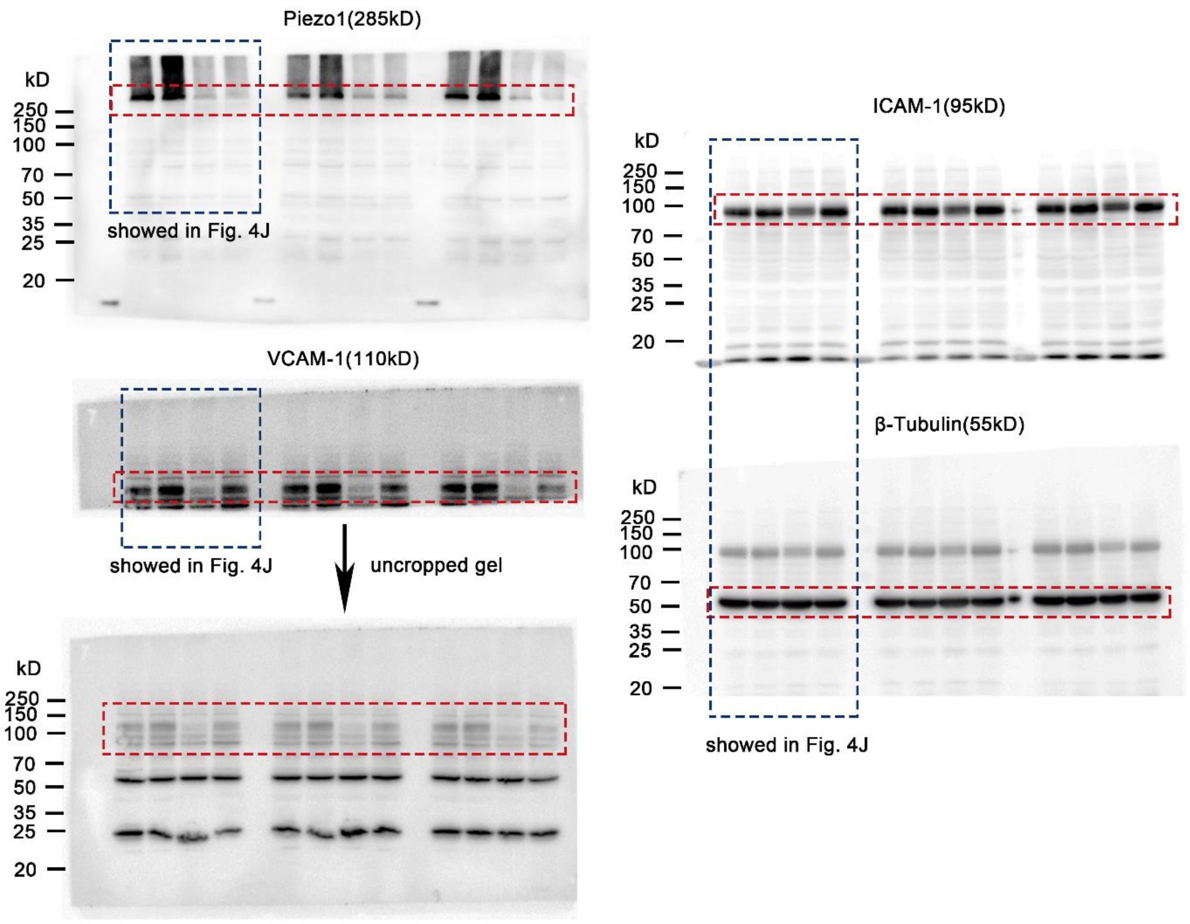

Full uncropped gels for Figure 5A

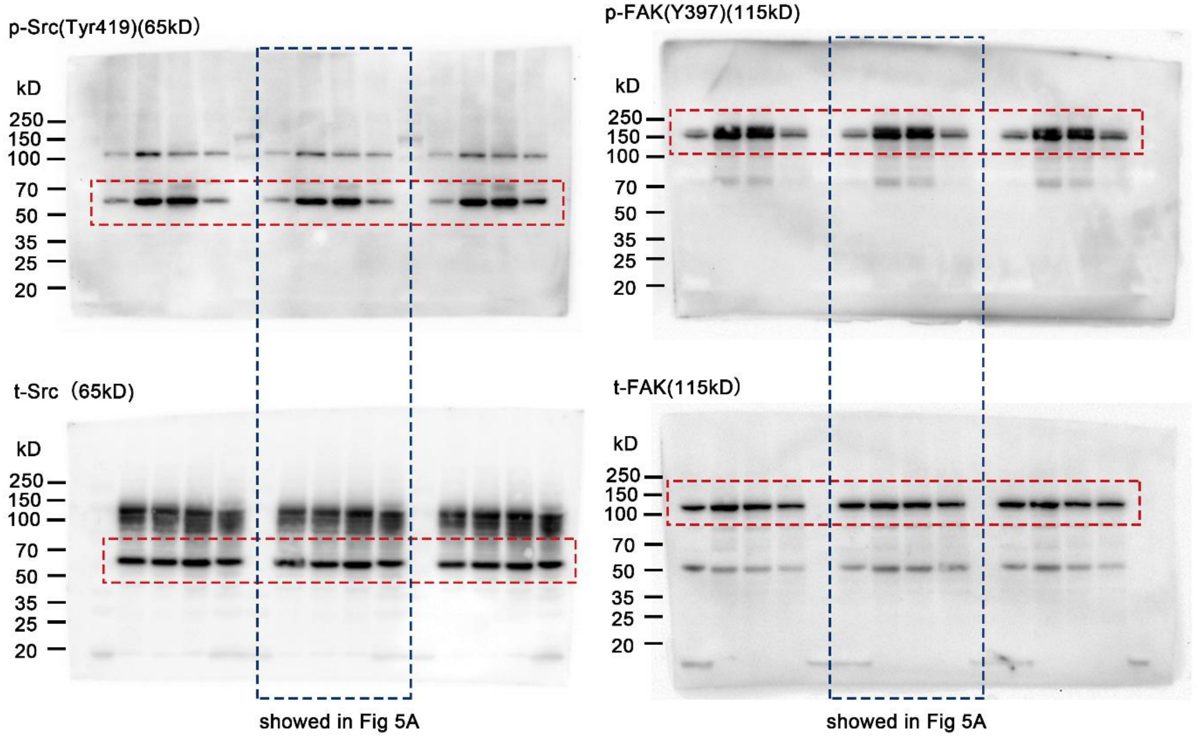

Full uncropped gels for Figure 5H

Input

FAK(119kD)

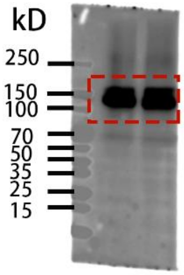

Input

Src(65)kD)

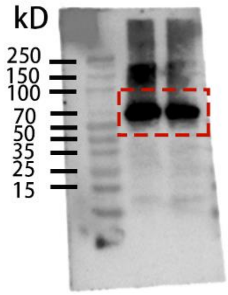

IP:FAK

IB:FAK(119kD)

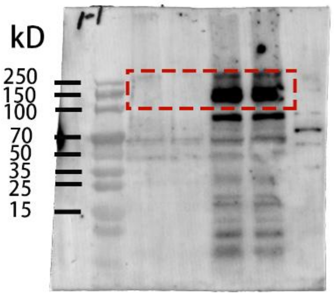

IP:Src

IB:FAK(119kD)

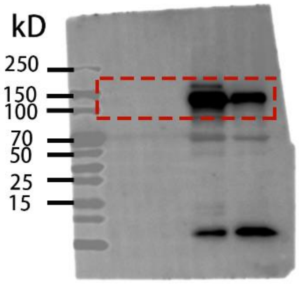

IP:FAK

IB:Src(65kD)

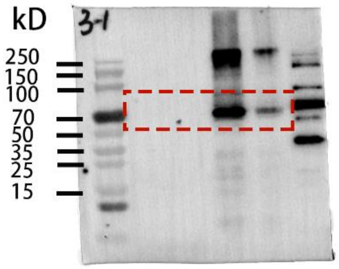

IP:Src

IB:Src(65kD)

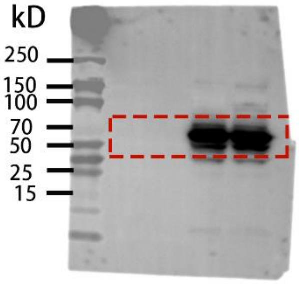

Full uncropped gels for Figure 5I

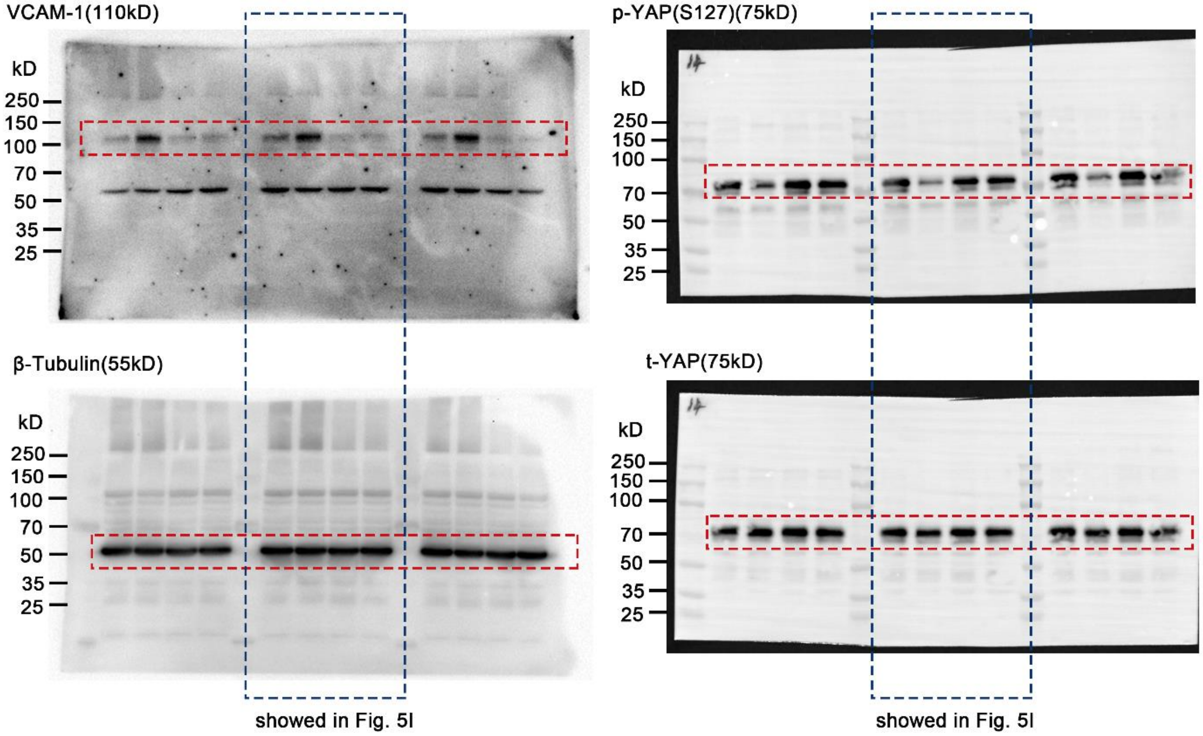

Full uncropped gels for Figure 6A

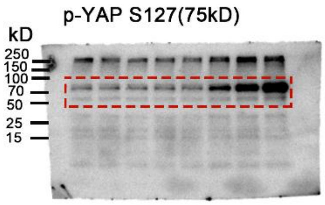

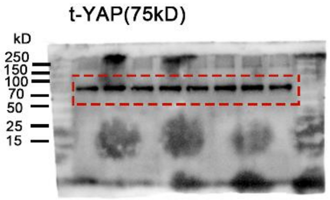

Full uncropped gels for Figure 6C

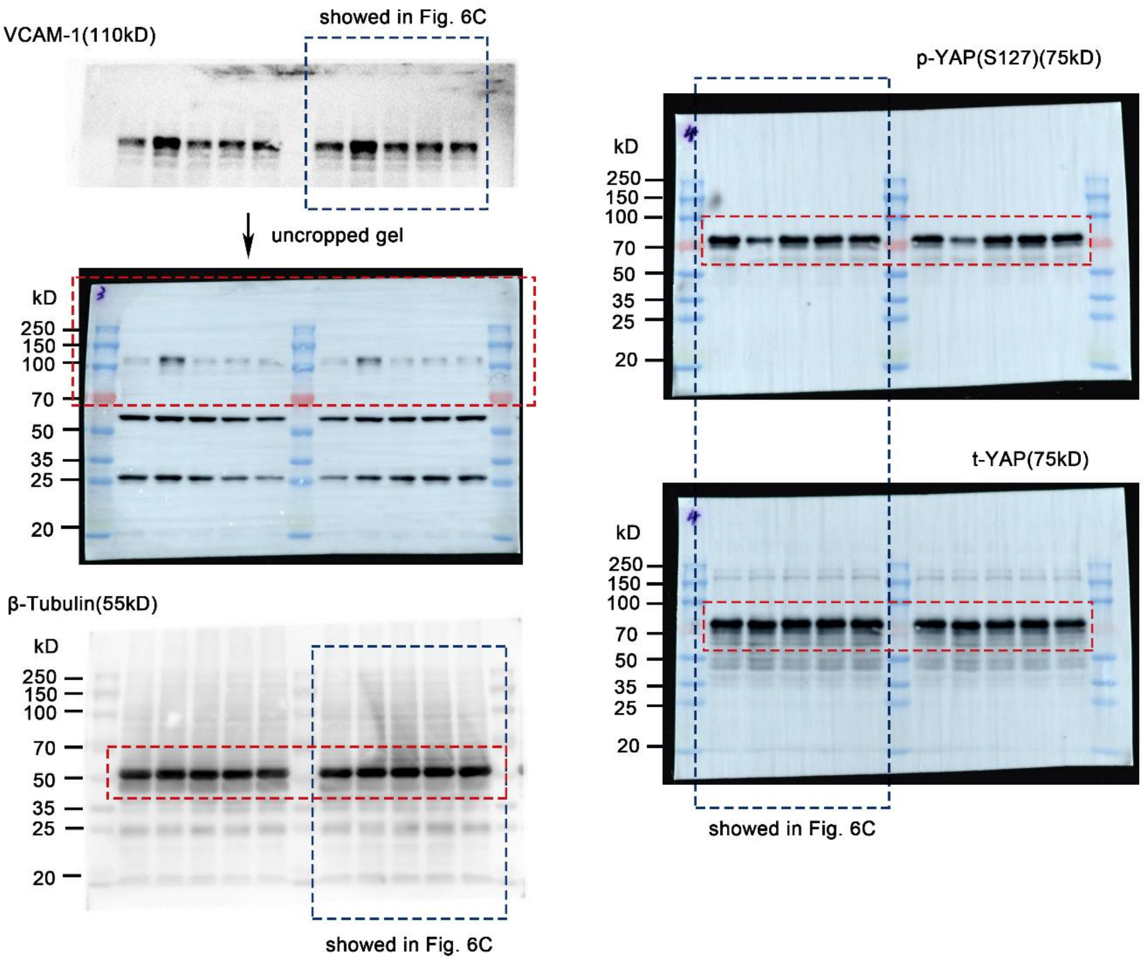

Full uncropped gels for Figure 6F

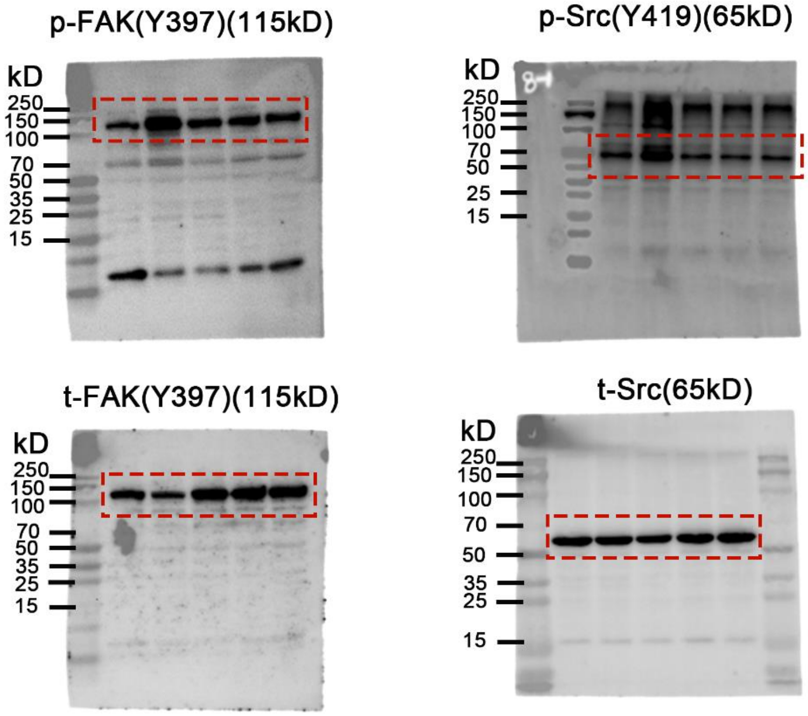

Full uncropped gels for Figure 6I

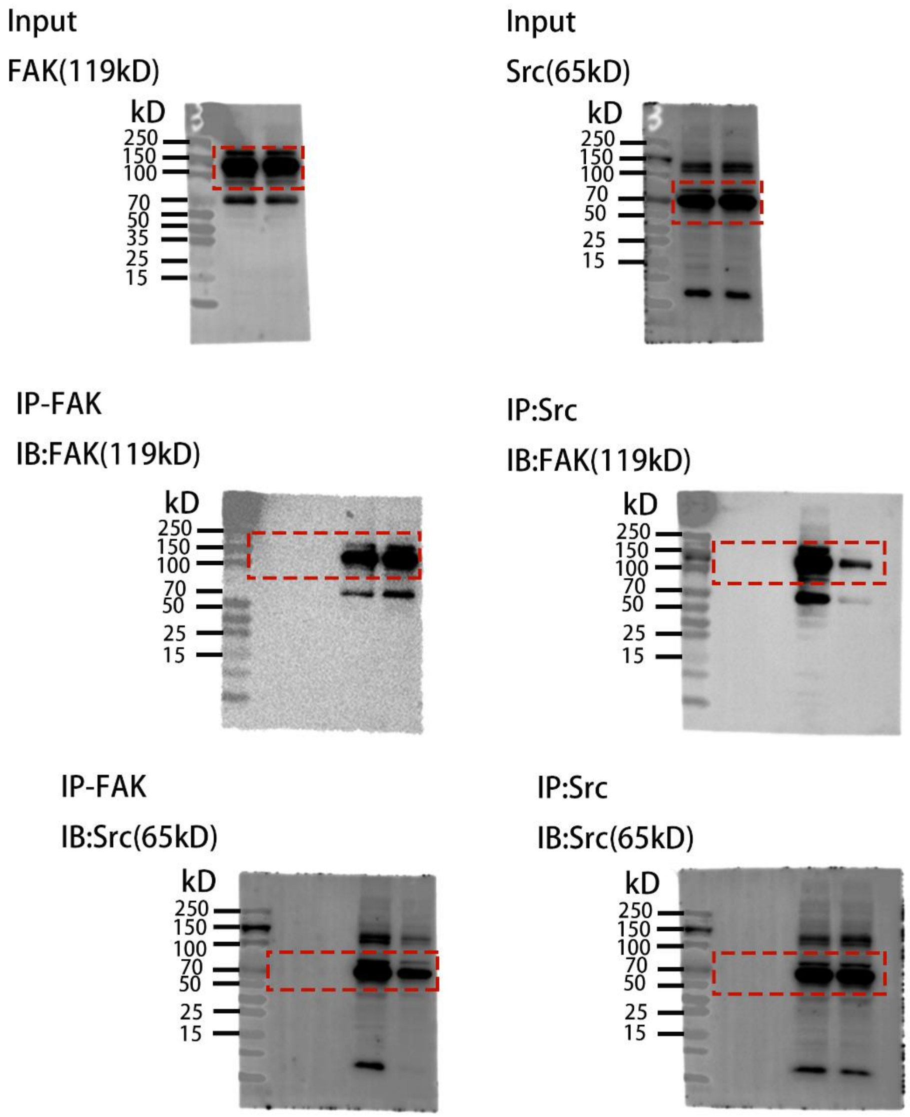

Supplement: Supplementary file 1 — Tables S1–S6 Figures S1–S6 Data S1 [file JAH3-13-e035558-s001.pdf]
